# Understanding the molecular mechanisms of odorant binding and activation of the human OR52 family

**DOI:** 10.1038/s41467-023-43983-9

**Published:** 2023-12-07

**Authors:** Chulwon Choi, Jungnam Bae, Seonghan Kim, Seho Lee, Hyunook Kang, Jinuk Kim, Injin Bang, Kiheon Kim, Won-Ki Huh, Chaok Seok, Hahnbeom Park, Wonpil Im, Hee-Jung Choi

**Affiliations:** 1 Department of Biological Sciences, Seoul National University, Seoul, 08826 Republic of Korea; 2 Department of Bioengineering, Lehigh University, Bethlehem, PA 18015 USA; 3 Department of Chemistry, Seoul National University, Seoul, 08826 Republic of Korea; 4 Brain Science Institute, Korea Institute of Science and Technology, Seoul, 02792 Republic of Korea; 5 Departments of Biological Sciences, Chemistry, and Computer Science and Engineering, Lehigh University, Bethlehem, PA 18015 USA; 6 Present Address: Perlmutter Cancer Center, NYU Langone Health, New York, NY USA

**Keywords:** Cryoelectron microscopy, Molecular modelling

## Abstract

Structural and mechanistic studies on human odorant receptors (ORs), key in olfactory signaling, are challenging because of their low surface expression in heterologous cells. The recent structure of OR51E2 bound to propionate provided molecular insight into odorant recognition, but the lack of an inactive OR structure limited understanding of the activation mechanism of ORs upon odorant binding. Here, we determined the cryo-electron microscopy structures of consensus OR52 (OR52_cs_), a representative of the OR52 family, in the ligand-free (apo) and octanoate-bound states. The apo structure of OR52_cs_ reveals a large opening between transmembrane helices (TMs) 5 and 6. A comparison between the apo and active structures of OR52_cs_ demonstrates the inward and outward movements of the extracellular and intracellular segments of TM6, respectively. These results, combined with molecular dynamics simulations and signaling assays, shed light on the molecular mechanisms of odorant binding and activation of the OR52 family.

## Abstract (web summary)

Limited insight existed on human odorant receptors (ORs). Here, the authors present structures of consensus OR52 in its inactive and active forms, shedding light on the activation mechanism of the OR52 family.

## Introduction

Humans perceive numerous odors from the environment. Olfaction, or the sense of smell, is initiated by the stimulation of odorant receptors (ORs) by odorants^1,2^. Humans have approximately 400 subtypes of ORs expressed on the surface of olfactory sensory neurons in a singular expression manner^3–5^. They recognize small-molecule odorants and induce the depolarization of olfactory sensory neurons^6,7^. The pairing of odorants and their cognate ORs has a combinatorial nature, allowing a person to smell far more types of odors than the number of ORs^1,6,8^.

ORs, which are members of class A G protein-coupled receptors (GPCRs), transduce downstream signals via the olfactory type G protein (G_olf_), which is highly homologous to G_s_^9,10^. Both G_olf_ and G_s_ stimulate adenylyl cyclase to produce cyclic AMP (cAMP), which activates the cAMP-dependent pathway^11^. Despite their classification within the class A GPCR subfamily, ORs do not possess all the conserved motifs that are characteristic of class A GPCRs. Notably, they lack the highly conserved residue W^6.48^ (superscript numbers refer to the GPCRdb numbering scheme^12^) and well-established P^5.50^-I^3.40^-F^6.44^ motif, which is critical for agonist-induced activation^13,14^.

Structural studies of mammalian ORs are challenging because of their limited surface expression in heterologous cells. Overexpression of ORs leads to robust endoplasmic reticulum retention and aggregation^15,16^. Extensive efforts have been made to overcome this problem, such as co-expression with receptor-transporting protein families, optimization of signal sequences, and large-scale mutagenesis screening^17–23^. The “consensus strategy”, which was previously applied to other proteins to improve thermostability^24,25^ has also been used to promote membrane trafficking of ORs, whereby the most frequent amino acids in a specific protein family are introduced to each residue position^26^. This approach has been successful in improving the surface expression of several human consensus ORs representing OR1, 2, 4, 5, 6, 10, 51, and 52 families while maintaining their odorant recognition abilities^23^.

Several subtypes of the human OR52 family, including OR52A5, OR52B2, OR52E1, OR52E8, and OR52L1, are known to recognize carboxylic acids^27,28^, and the consensus OR52 (OR52_cs_), representing 26 members of the human OR52 family (Supplementary Fig. 1), is also responsive to carboxylic acid odorants^23^. Although the recently published structure of propionate (PPI)-bound OR51E2 has provided insights into the molecular basis for the binding of carboxylic acid odorants^29^, the molecular mechanism of OR activation by odorant binding remains unclear as there is no OR structure in an inactive state for comparison with the active structure.

Herein, we aimed to elucidate the mechanisms of odorant binding and activation of the OR52 family using OR52_cs_ as a representative. By comparing the ligand-free (apo) and octanoate (OCA)-bound states of OR52_cs_, we discovered a unique mechanism involving the large inward movement (7.4 Å) of the extracellular segment of TM6 upon OCA binding, in contrast to the 2-3 Å TM6 movement of non-olfactory class A GPCRs upon agonist binding. Our structural study, together with sequence analysis, mutagenesis studies, and molecular dynamics (MD) simulations, revealed a distinctive activation mechanism of the human OR52 family in response to carboxylic acid odorants with long hydrocarbon tails.

## Results

### Structure of the OCA–OR52_cs_–G_s_–Nb35 complex

**Fig. 1:**
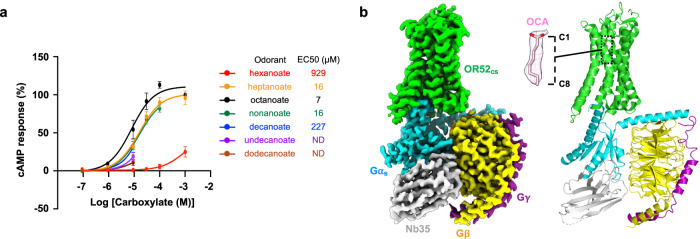
Overall structure of the OCA–OR52_cs_–G_s_–Nb35 complex. **a** Dose-response curves of OR52_cs_ for carboxylic acid odorants of different lengths; hexanoate (red), heptanoate (orange), octanoate (OCA) (black), nonanoate (green), decanoate (blue), undecanoate (purple), and dodecanoate (brown). Each data point represents the mean ± standard error of the mean (S.E.M.) from *n* = 3 independent experiments except for OCA (*n* = 5 independent experiments). **b** Cryo-EM map (left) and model (right) of the OCA–OR52_cs_–G_s_–Nb35 complex. OR52_cs_, Gα_s_, Gβ_1_, Gγ_2_, and Nb35 are colored green, cyan, yellow, magenta, and light-gray, respectively. OCA is shown as pink sticks, with the surrounding density map displayed at a threshold of 0.336 in a zoomed view.

To identify the optimal odorant for OR52_cs_, we performed a downstream signaling assay on OR52_cs_. Among the various lengths of carboxylic acid odorants tested (ranging from hexanoate to dodecanoate), OCA, which showed the lowest EC_50_ value, was selected for our structural study (Fig. 1a). Subsequently, we verified the substitutability of Gα_olf_ with Gα_s_ in its interaction with OR52_cs_ by bioluminescence resonance energy transfer (BRET) assays and structure determination of Gα_olf_ in the GTPγS-bound state at 2.9 Å resolution (Supplementary Fig. 2 and Supplementary Table 1). Structural comparison highlighted the high similarity between Gα_olf_ and Gα_s_ particularly in the C-terminal α helix, which is a major GPCR binding site (Supplementary Fig. 2). Finally, we purified the complex of full-length OR52_cs_, G_s_, and Nb35 (the complex-stabilizing nanobody^30^) in the presence of OCA, and solved the cryo-EM structure of the OCA–OR52_cs_–G_s_–Nb35 complex at a global resolution of 2.97 Å (Fig. 1b, Supplementary Fig. 3 and Supplementary Table 2). After local refinement of the receptor, a 3.09 Å resolution receptor-focused map was obtained, in which most residues of OR52_cs_ were well resolved and a clear density of OCA was identified within the transmembrane pocket (Fig. 1b).

Similar to other previously reported GPCR–G_s_ structures, the C-terminus α5 helix of Gα_s_ is inserted into the cytoplasmic cavity of OR52_cs_ (Supplementary Fig. 4). The OR-conserved VAIC sequences, DRY motif, and several residues in the intracellular loop (ICL) regions (E59^12.52^, R133^34.52^, Y134^34.53^, and S232^ICL3^) are involved in interactions with Gα_s_ (Supplementary Fig. 4). Multiple sequence alignment showed that most of these residues are highly conserved across human ORs, suggesting the conserved binding interface between OR and Gα_s_/Gα_olf_ (Supplementary Fig. 4).

### Overall structural features of OR52_cs_

**Fig. 2:**
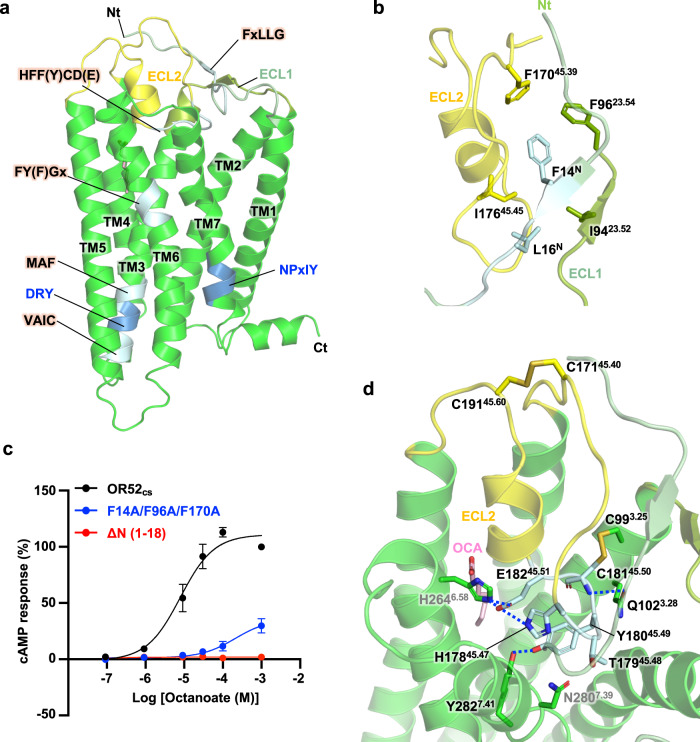
Structural features of OR52_cs._ **a** Overall structure of OR52_cs_ highlighting conserved motifs. The OR-specific conserved motifs are colored in light blue, and the conventional motifs present in class A GPCRs (DRY and NPxIY motifs) are colored in blue. N-tail, ECL1, and ECL2 are colored in pale green, split pea, and yellow, respectively. The TM helices and structural motifs are labeled. **b** Detailed interactions among the N-tail, ECL1, and ECL2. Residues interacting with F14^N^ and L16^N^ in the conserved FxLLG motif are shown as sticks. **c** Dose-dependent cAMP response curves of the N-tail deletion mutant (ΔN(1–18)) and the F14A/F96A/F170A mutant. Each data point represents the mean ± standard error of the mean (S.E.M.) from *n* = 3 independent experiments. **d** Interactions of ECL2 with TMs 3, 6, and 7. The two disulfide bonds, C99^3.25^-C181^45.50^ and C171^45.40^-C191^45.60^, are shown as sticks. Residues in the conserved HTYCE motif are colored in light blue. Highly conserved residues in ORs are labeled in black, whereas less conserved residues are labeled in gray.

OR-specific conserved motifs, such as the FxLLG motif in the N-tail and the HFF(Y)CD(E) motif in ECL2^2,31^, are essential for maintaining the structural integrity of OR52_cs_ (Fig. 2a). The residue F14^N^ within the FxLLG motif forms an aromatic interaction network with two other phenylalanine residues in ECL1 and ECL2 (F96^23.54^ and F170^45.39^) (Fig. 2b). These three Phe residues are highly conserved in human ORs (F/Y) (Supplementary Fig. 5). Substitution of these residues with alanine resulted in a significantly reduced cAMP response upon OCA treatment (Fig. 2c). Another conserved residue, L16^N^, forms a stable interaction network with I94^23.52^ and I176^45.45^, which are also conserved in human ORs (Fig. 2b). Notably, the hydrophobic interaction network mediated by the N-tail appears to be crucial for functional OR52_cs_ expression as demonstrated by the significant decrease in surface expression and, consequently, loss of downstream signaling in the N-terminally truncated OR52_cs_ mutant (Fig. 2c and Supplementary Table. 3). The ECL2 region of OR52_cs_ containing the HTYCE motif is embedded within the transmembrane pocket formed by TMs 3, 4, 6, and 7 (Fig. 2d). This U-shaped conformation of ECL2 is stabilized by the HTYCE motif-mediated interactions: H178^45.47^–H264^6.58^, T179^45.48^–N280^7.39^, Y180^45.49^–Y282^7.41^, and E182^45.51^–H264^6.58^, all of which are conserved in the OR52 family (Fig. 2d and Supplementary Fig. 5). The ECL2 conformation is further stabilized by two disulfide bonds (C171^45.40^– C191^45.60^ and C181^45.50^– C99^3.25^) (Fig. 2d and Supplementary Fig. 6). This TM-embedded conformation of ECL2 is likely to impede odorant entry into the central ligand-binding pocket from the extracellular side. Details of odorant binding to OR52_cs_ are discussed below.

When comparing our structure with the recently published structure of OR51E2, we observed very similar overall structures between them, the consensus OR and the native OR, with a RMSD of 1.5 Å for 289 Cα atoms (Supplementary Fig. 6)^29^. Furthermore, our structural analysis of OR52_cs_, in combination with extensive sequence analysis of ORs, suggested that the interaction network observed in this structure is conserved in the human OR52 family (Supplementary Fig. 1), highlighting that the OR52_cs_ structure presented here provides a comprehensive view of the overall architecture of the human OR52 family.

### Odorant recognition by OR52_cs_

**Fig. 3:**
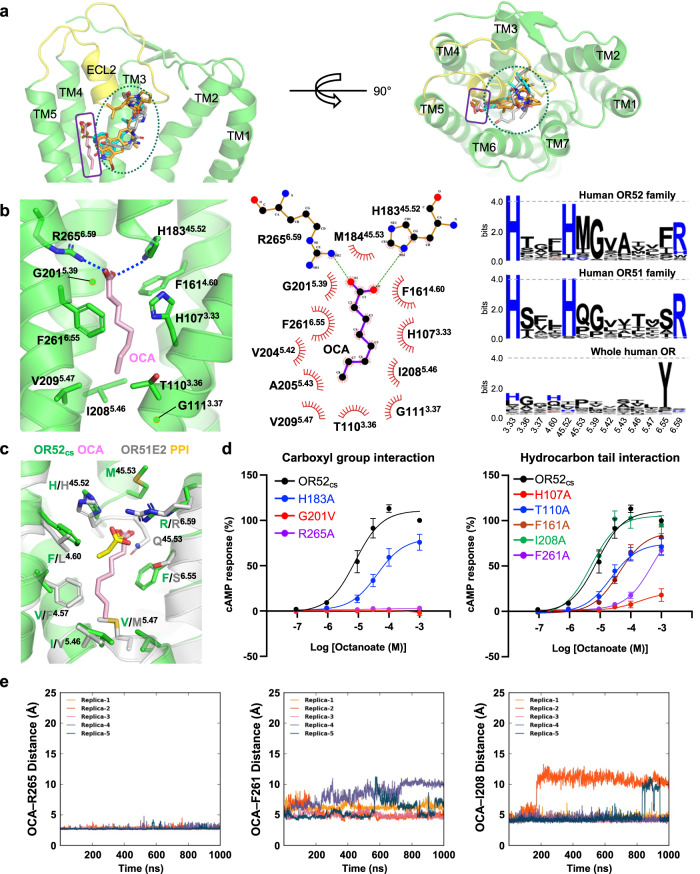
Odorant binding site of OR52_cs._ **a** Comparison of ligand-binding sites in OR52_cs_, OR51E2, and non-olfactory class A GPCRs. Agonists bound to OR51E2 (PDB: 8F76), β_2_-adrenergic receptor (PDB:3SN6), dopamine D2 receptor (PDB:6VMS), and μ-opioid receptor (PDB:6DDF) are shown as yellow, cyan, orange, and gray sticks, respectively, and OCA are shown as pink sticks. The orthosteric ligand-binding site and OCA binding site are indicated by blue ellipse and purple square, respectively. The OR52_cs_ structure is shown in cartoon representation, and TM6 and TM7 are removed in the side view on the left panel for clarity. **b** OCA and its interacting residues within 4.5 Å of OCA are shown as sticks, and the Cα atom of Gly is represented as a green ball. The polar interactions are indicated as dashed blue lines (left). The interaction between OCA and OR52_cs_ was analyzed and visualized using LigPlot+ v.2.2.7 (middle). Sequence conservation of the residues constituting the odorant binding pocket in the human OR52 family, OR51 family, and whole ORs is depicted using WebLogo 3 (right). **c** Structural alignment of OR52_cs_ with OR51E2. OR51E2 is shown as light-gray, and PPI is shown as a yellow stick. Residues within 4.0 Å of the odorants are shown as sticks. **d** Dose-dependent cAMP response curves of OR52_cs_ (black) and mutants by OCA treatment. The EC_50_ values for each curve are summarized in Supplementary Table 3. The mean ± S.E.M. from *n* = 5 independent experiments (OR52_cs_) and *n* = 3 independent experiments (mutants) are shown as symbols and error bars, respectively. **e** All-atom MD simulations of the OCA–OR52_cs_–G_s_ system. The distances between OCA and R265^6.59^, F261^6.55^, and I208^5.46^, respectively, were plotted over the 1 µs simulation time for five replicas.

The odorant-binding pocket in OR52_cs_ is formed by TMs 3, 4, 5, and 6 and distantly from TMs 1, 2, and 7, unlike the orthosteric ligand-binding pockets of most class A GPCRs (Fig. 3a). Notably, the TM-embedded ECL2 of OR52_cs_ partially overlaps with this orthosteric ligand-binding site of non-olfactory class A GPCRs (Fig. 3a). In the structure of PPI-bound OR51E2, the odorant binding site is located similarly to that in OR52_cs_ (Fig. 3a), supporting the notion that the architecture of the odorant-binding pocket in OR52_cs_ is representative of the carboxylate-recognizing OR52 family.

OCA contains a negatively charged carboxyl group, like PPI, but harbors a longer hydrophobic hydrocarbon moiety (Fig. 3b). The carboxyl group of OCA forms electrostatic interactions with R265^6.59^ and H183^45.52^ of OR52_cs_ (Fig. 3c). The importance of R265^6.59^ for OCA binding was demonstrated by the complete loss of downstream signaling in the R265^6.59^A mutant (Fig. 3d). In addition, all-atom MD simulations showed that the interaction distance between the carboxyl group of OCA and R265^6.59^ is maintained within a distance of an average of 2.8 Å in 1 μs simulations (Fig. 3e). Notably, R265^6.59^ is highly conserved in human OR51/52 families, recognizing carboxylic acids as odorants^32–34^, but not in other OR families (Fig. 3b), suggesting that this Arg residue is responsible for the specific recognition of carboxylic acid odorants in OR51/52 members. In addition, octanol, which contains a hydroxyl group instead of a carboxyl group at the C1 position, failed to activate OR52_cs_, highlighting the essential role of the carboxyl group of the odorant in OR52_cs_ activation (Supplementary Fig. 7). The conserved G201^5.39^ in the OR51/52 families plays a critical role in constituting the odorant binding site. In both our structure and the PPI-OR51E2 structure, the Cα atom of G^5.39^ is within 4 Å of the carboxyl oxygen atom of the odorants (Fig. 3b), indicating that a side chain at this position can cause steric clashes with the carboxyl group of the odorant. Indeed, the replacement of Gly with Val (G201^5.39^V) in OR52_cs_ resulted in the loss of OCA signaling (Fig. 3d).

The hydrocarbon moiety of OCA is surrounded by hydrophobic residues. In particular, F261^6.55^ forms an extensive hydrophobic interaction network with multiple carbon atoms of OCA, and H107^3.33^ forms van der Waals contacts with the C3 and C5 carbons of OCA (Fig. 3b). The substitution of these residues with Ala significantly reduced downstream signaling (Fig. 3d and Supplementary Table. 3). At the bottom of the ligand binding site, T110^3.36^, G111^3.37^, I208^5.46^, and V209^5.47^ are closely located within a distance range of 3.5 to 4.5 Å from the C8 atom of OCA, forming van der Waals contacts with each other (Fig. 3b). Unlike the stable contact between the carboxyl group of OCA and R265^6.59^, the distances between the hydrocarbon tail of OCA and its interacting hydrophobic residues showed some fluctuations in the 1 μs all-atom MD simulations (Fig. 3e). This observation may be attributed to the fact that the ligand-binding pocket is not specifically designed only for OCA, but can also accommodate other odorants such as heptanoate and nonanoate (Fig. 1a).

A comparison of the odorant binding pockets of OR52_cs_ and OR51E2 indicates that while the carboxyl groups of PPI and OCA interact similarly with the conserved R^6.59^ in each OR, the orientation of the hydrocarbon tails is slightly different in the two structures. In OR51E2, L158^4.60^ provides contact for the hydrocarbon tail and stabilizes the slightly lateral orientation of PPI. In contrast, the corresponding residue in OR52_cs_ is F161^4.60^, which restricts the lateral orientation of OCA (Fig. 3c). The downward orientation of OCA tail is stabilized by extensive van der Waals contacts with F261^6.55^, whereas the corresponding residue S258^6.55^ of OR51E2 participates in polar interaction with the carboxyl group of PPI (Fig. 3c).

In a previous report, the F155^4.57^A mutation of OR51E2 showed selectivity for longer-chain fatty acids^13^. Our structural analysis suggested that the residue at position 5.47 may also affect the selectivity for fatty acid chain lengths. Indeed, F155^4.57^ and M206^5.47^ in OR51E2 are replaced with smaller side chains in OR52_cs_, V158^4.57^ and V209^5.47^, respectively (Fig. 3c). Our cAMP assay showed that the V158^4.57^F mutation of OR52_cs_ greatly reduced the downstream signal for OCA and enhanced selectivity for heptanoate (Supplementary Fig. 8). Double mutation of V158^4.57^F/V209^5.47^M of OR52_cs_ showed increased responses to shorter chain fatty acids such as hexanoate, but the preference for OCA was maintained (Supplementary Fig. 8). This result implies that the selectivity for odorant chain length is determined by the combinatorial effect of amino acids constituting the odorant-binding pocket, rather than being dictated by a single residue or two.

### Structure of apo state OR52_cs_

The structure of OCA-bound OR52_cs_ revealed that it would be difficult for an odorant with a long hydrophobic tail to approach the TM pocket from the extracellular side without undergoing large conformational changes as the binding pocket is occluded in the active structure (Supplementary Fig. 9). To understand the mechanism by which a fatty acid odorant with a long hydrocarbon tail reaches the ligand-binding pocket, we aimed to determine the structure of OR52_cs_ in the apo state, without a bound ligand. For the structural study, we utilized a fiducial marker strategy, which has previously been used to determine the apo state structure of FZD5 using cryo-EM^35^. The four-helical bundle fusion partner of thermostabilized apocytochrome b562 (bRIL) was inserted into ICL3 of OR52_cs_ (OR52_cs_-bRIL). This chimeric protein, which showed neither basal activity nor OCA-induced activity, was purified in complex with a Fab recognizing bRIL for the cryo-EM study (Supplementary Figs. 10, 11).

**Fig. 4:**
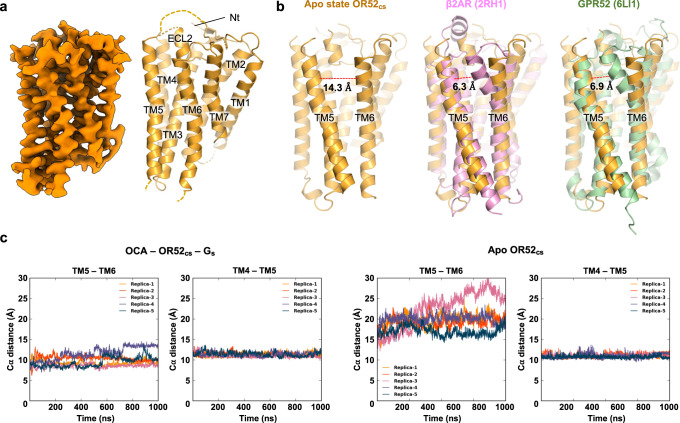
Structural characteristics of the apo state OR52_cs_. **a** Cryo-EM map and model of the apo state OR52_cs_ are shown. **b** The structures of carazolol-bound β_2_AR (PDB: 2RH1, pink)^42^ and apo state GPR52 (PDB: 6LI1, palegreen)^37^ are aligned against TM5 of the apo structure of OR52_cs_ (orange). The distance between the extracellular regions of TM5 and TM6 was measured between Cα atoms of residues at positions 5.40 and 6.56 in each structure, and is displayed with a red dashed line. **c** All-atom MD simulations were performed for OCA–OR52_cs_–G_s_ (left) and apo state OR52_cs_ (right). The fluctuations in the distance between TM5(L202^5.40^) and TM6(L262^6.56^), and the distance between TM4(L164^4.63^) and TM5(K195^5.33^) are plotted during the simulation time. Five replicas were simulated independently for each system. The simulation time is 1 µs for both systems.

To date, only a few class A GPCR structures have been reported in the apo state, such as structures of rhodopsin and constitutively active GPR52^36,37^, likely due to the structural flexibility of class A GPCRs in the absence of ligand^38–40^. With a large dataset, extensive 3D classification, and local refinement of the receptor, we successfully obtained a cryo-EM map at approximately 4 Å for model building of the apo state OR52_cs_ (Fig. 4a, Supplementary Fig. 10 and Supplementary Table 2).

The apo structure of OR52_cs_, which aligned with the AlphaFold2 prediction model^41^ with a RMSD of 1.1 Å for 242 Cα atoms (Supplementary Fig. 12), reveals an unprecedented feature— a large opening between the extracellular parts of TM5 and TM6 that is not seen in other class A GPCRs in their inactive states^42^. Indeed, the distance between L202^5.40^ and L262^6.56^ reaches 14 Å in the apo state of OR52_cs_, compared to 6–7 Å in most class A GPCRs (Fig. 4b). All-atom MD simulations of the apo state OR52_cs_ showed considerable fluctuations in the distance between TM5 and TM6, whereas the distance between these helices remained stable in the active state (Fig. 4c). In the apo state, the extracellular segment of TM6 (residues 255-266) makes only a few contacts with TM7 residues, whereas the intracellular segment of TM6 (residues 237-254) forms close contacts with residues on TM5. C243^6.37^ and H246^6.40^ form close contacts with Y220^5.58^, and I250^6.44^ interacts with I216^5.54^ (Supplementary Fig. 11). In the middle of TM6, Y254^6.48^ forms hydrogen bonds with S114^3.40^ (Supplementary Fig. 11). These interactions stabilize the inactive structure, where the G protein binding pocket is closed (Supplementary Table. 4).

### Structural changes in OR52_cs_ upon odorant binding

**Fig. 5:**
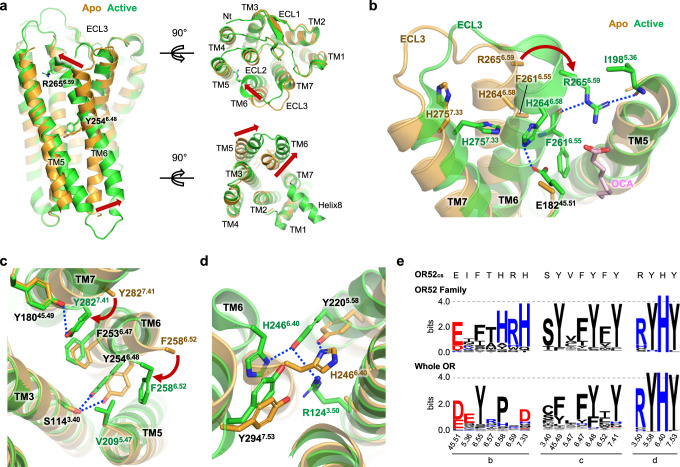
Comparison of the apo and active states of OR52_cs._ **a** An overlay of the apo (orange) and active (green) states of OR52_cs_ is presented in the side (left), extracellular (upper right), and intracellular (bottom right) views, with movements of the TMs highlighted by red arrows. Y254^6.48^ of the FY^6.48^xP motif that act as a pivot and R265^6.59^ are shown as sticks. **b** Residues participating in interactions that stabilize the extracellular segments of TM6 and TM7 are shown as sticks. Polar interactions are represented as dashed blue lines. The rotation of R265^6.59^ upon activation is indicated by a red arrow. The side chains of F261^6.55^, H264^6.58^, and R265^6.59^ are unresolved in the apo structure. **c** Interactions between F258^6.47^ and V209^5.47^ and between Y282^7.41^ and Y180^45.49^, which are observed only in the active state, are shown, with the rotation of F258^6.47^ and Y282^7.41^ upon activation, indicated by red arrows. OCA is not shown for clarity. **d** The interaction network of TM3-TM6-TM7 formed upon receptor activation is shown. **e** Conservation of key residues stabilizing the active conformation, highlighted in (**b**–**d**), is depicted using WebLogo 3.

Comparison of the apo and active structures of OR52_cs_ provided insights into conformational changes upon receptor activation (Fig. 5a). Notably, TM6 underwent substantial conformational changes, with an inward movement at the extracellular end (7.4 Å shift of Cα^6.59^ atom) and an outward movement at the intracellular end (9.0 Å shift of Cα^6.34^ atom) upon OCA binding, acting Y254^6.48^ of the conserved FYxP motif as the pivot (Fig. 5a). In addition, TM6 exhibited a 2–3 Å upward shift. As the TM6 conformation changes, the interaction network that stabilizes the inactive conformation is rearranged.

Notably, R265^6.59^, a critical residue for OCA binding, exhibits a 7.4 Å inward movement towards the odorant-binding pocket upon OCA binding, and its inward conformation was stabilized by multiple interactions in the active structure. R265^6.59^, engaged with the carboxyl group of OCA, forms polar interactions with the carbonyl groups of I198^5.36^ and F261^6.55^, and van der Waals interactions with I198^5.36^ (Fig. 5b). The inward conformational shift of the extracellular part of TM6 is further stabilized by the formation of hydrogen bonds of H264^6.58^ with E182^45.51^ and H275^7.33^ (Fig. 5b). F258^6.52^ underwent an inward movement to form a hydrophobic interaction with V209^5.47^ (Fig. 5c), although the side chains of both residues were not well-resolved in the apo state. Upon OCA binding, Y254^6.48^ of the FYxP motif shifted upward by approximately 2.8 Å, while retaining its hydrogen bond with S114^3.40^ (Fig. 5c). F253^6.47^ also did not undergo a large conformational change except for a 2.4 Å upward movement, and interacted with Y282^7.41^, which underwent a large inward movement upon activation (Fig. 5c). In turn, Y282^7.41^ forms a polar contact with Y180^45.49^ of the conserved HS(T)YCD(E) motif (Fig. 5c). At the intracellular part of TM6, H246^6.40^ undergoes a 4.5 Å outward movement with a downward rotamer change, to form a van der Waals contact with Y294^7.53^ of the NPxIY motif (Fig. 5d). Y220^5.58^ moved inward by 3.4 Å, to form a hydrogen bond with R124^3.50^ in the DRY motif (Fig. 5d). The importance of these interactions for OR52_cs_ activation was demonstrated by reduced efficacy or potency of Ala mutations of each interacting residue (Supplementary Fig. 13). Given the high conservation of these interacting residues in the OR52 family, it is plausible that these conformational changes upon activation could occur in native human OR52 members (Fig. 5e and Supplementary Fig. 1).

**Fig. 6:**
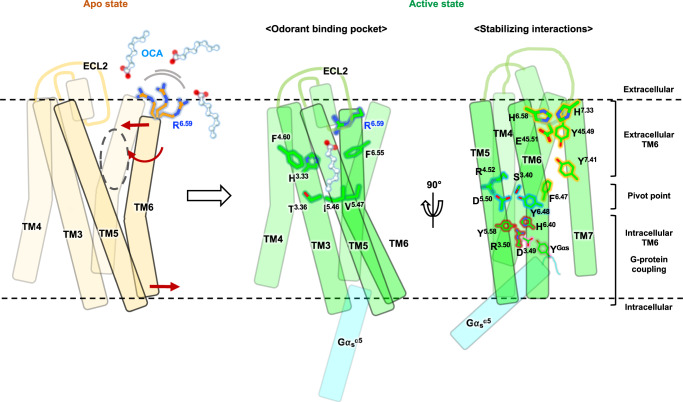
Mechanism of OR52_cs_ activation by OCA binding. Schematic representation of the activation mechanism of OR52_cs_ upon OCA binding. In the apo state (left, light yellow), there is a wide opening between TM5 and TM6, possibly acting as an entrance for OCA. The highly conserved R^6.59^ on the extracellular end of TM6 is flexible, represented as multiple conformations. Inward and outward movements of the extracellular and intracellular regions of TM6 and TM6 rotation upon OCA binding are indicated by red arrows. In the active state (green), OCA-mediated interactions in the odorant-binding pocket (middle), and distinct interaction networks that stabilize the active conformation (right) are presented. The active conformation is further stabilized by interactions between OR52_cs_ and G protein (cyan). OCA molecules are shown in the ball-and-stick model. Figure was generated using ChimeraX and Powerpoint.

The observation of a large opening between TM5 and TM6 in the apo structure prompted us to hypothesize that an odorant with a long hydrophobic tail could approach the odorant binding pocket through this opening. To gain a deeper understanding of the odorant binding mechanism in OR52_cs_, we performed extensive all-atom MD simulations. Initially, we attempted to observe OCA entering the odorant binding pocket of OR52_cs_ using the apo structure as a starting model. However, this was not successful, possibly because our simulation timescale was not sufficiently long to capture such events. Therefore, we conducted all-atom MD simulations of the OCA-bound active state OR52_cs_ (with and without G_s_) to understand how OCA exits the pocket. In the 20 µs simulation without G_s_, we observed that the extracellular part of TM6 moved outward after 1 µs simulation time (Supplementary Fig. 14). As a result, OCA escaped from the pocket through this wide opening between TM5 and TM6, a characteristic structural feature of the apo-state OR52_cs_. In this state, R265^6.59^ and F261^6.55^, which initially pointed toward the odorant binding pocket, reoriented toward the lipid bilayer due to the TM6 rotation. Even when OCA was out of the pocket, R265^6.59^ still captured the carboxyl group of OCA whereas F261^6.55^ lost interactions with the hydrocarbon tail of OCA. Interestingly, OCA captured by R265^6.59^ re-entered the TM pocket around 8 µs simulation time, although it was not properly positioned in the odorant binding pocket because of the lipid entering the TM pocket (Supplementary Fig. 14). Combining these observations, a probable OCA binding mechanism can be inferred: OCA is first captured by R265^6.59^, and then enters the odorant binding pocket of the apo-state OR52_cs_. The conformational changes of OR52_cs_ upon OCA binding stabilizes the active structure of OR52_cs_, which is further potentiated by G protein binding (Fig. 6). Intriguingly, during the same simulation timescale, OCA in OR52_cs_ bound to G_s_ did not escape the receptor.

## Discussion

In this study, we present a structural analysis of the OR52 family using a consensus strategy (represented by OR52_cs_) to overcome the low expression of ORs in heterologous cells. Our research demonstrates the structural characteristics of the odorant-binding pocket of OR52_cs_, which can accommodate carboxylic acids with carbon lengths of 7–9. Furthermore, we unveiled the odorant binding mechanism by extensive all-atom MD simulations and a comparative study of the apo and active structures. We highlight the importance of conserved residues in the OR52 family, particularly R^6.59^, in recognizing the carboxyl group of OCA, and hydrophobic residues, F^4.60^ and F^6.55^, in interacting with the hydrocarbon tail of OCA.

Based on our OR52_cs_ structure, we inferred the odorant specificity of some OR52 family members, including OR52N2, OR52N5, OR52E4, OR52E5, and OR52L1, via a sequence analysis of the amino acids constituting the odorant binding pocket (Supplementary Table. 5). However, our attempts to verify odorant specificity using cAMP assays with fatty acids ranging from pentanoate to undecanoate were unsuccessful under our experimental conditions, likely due to their limited surface expression (Supplementary Fig. 15). As OR52L1 was previously reported to be responsive to pentanoate^28^, we conducted computational modeling of pentanoate-bound OR52L1 using our OCA–OR52_cs_ structure as a template. This model showed that most residues involved in odorant binding are well conserved in both structures (Supplementary Fig. 15). Regarding OR52E5, an orphan OR, our computational simulations for various lengths of fatty acids imply that the optimal range could be from octanoate to decanoate, based on the stability of the odorant-binding pocket during 200 ns all-atom MD simulations (Supplementary Fig. 15). Although these computational models should be experimentally verified in the future, we believe that our OR52_cs_ structure can be used for computational modeling and virtual screening to deorphanize orphan OR52 family members^43^.

The apo structure of OR52_cs_ in this study provides an interesting structural feature of OR52_cs_ in the absence of ligand, which is distinct from previously reported class A GPCR structures in the apo- or inactive states. Notably, the extracellular segment of TM6 is located 14 Å away from TM5, resulting in a wide opening between TM5 and TM6. This unique feature suggests that the hydrophobic tail of OCA in the lipid bilayer may access the transmembrane pocket of OR52_cs_ through this opening. Upon OCA binding, we observe substantial inward and outward movements of the extracellular and intracellular parts of TM6, respectively, with Y254^6.48^ serving as a pivot point. These conformational changes are stabilized by the interactions between OCA and the transmembrane pocket of OR52_cs_, as well as the interactions enabled by the repositioning of F258^6.52^, H264^6.58^, R265^6.59^, and Y282^7.40^. Subsequently, G protein coupling at the cytoplasmic region further stabilizes the active conformation of OCA-bound OR52_cs_. Importantly, this study provides direct evidence of substantial TM6 conformational changes upon activation, distinguishing it from the established activation mechanism of class A GPCRs. It is noteworthy that the amino acids involved in the activation process are highly conserved in the OR51/52 families but not in all ORs, implying a potential diversity of mechanisms in ORs with varying odorant types.

Extensive all-atom MD simulations suggest that hydrophobic odorants may enter and exit the odorant pocket through an opening between TM 5 and TM6. In the absence of G_s_, MD simulation of OCA-bound OR52_cs_ revealed the escape of OCA from the pocket in 2 µs through the opening between TM5 and TM6, and concomitantly, the intrusion of phospholipids into this space, which restricts the inward movement of TM6 (Supplementary Fig. 16). In a longer simulation time even after OCA completely escapes the odorant pocket, we could observe dynamic exchange of phospholipids, which leads to a dynamic TM5-TM6 gap, providing a pathway for OCA to access the binding pocket. When G_s_ is engaged, the OCA-bound active conformation of OR52_cs_ is maintained in our simulation time (10 µs), preventing phospholipid intrusion. This indicates that both odorant binding and G protein coupling are required for stabilizing OR52_cs_ in its fully active conformation, which is capable of transmitting downstream signals. While our MD simulation data, together with the hydrophobic nature of the OCA tail supports the plausibility of the lateral entry pathway, further studies are needed to understand the odorant entry mechanism. Of note, within the in vivo system, odorant-binding proteins that transport hydrophobic odorants are present within nasal mucus^44,45^.

Overall, this study provides valuable insights into the activation mechanism and odorant specificity of the OR52 family, and paves the way for further experimental and computational studies to unravel the mechanisms underlying OR function.

## Methods

### Construct design

Full-length human Gα_s_ (short isoform) with the C3S mutation was cloned into the pFastBac HT B vector. For the Gβ and Gγ subunits, full-length wild-type human Gβ_1_ and Gγ_2_ were cloned into the pFastBac Dual expression vector. N-terminal 6xHis-tag followed by a human rhinovirus 3C (HRV 3C) protease site was introduced into the plasmids harboring Gα_s_ and Gβ_1_ for affinity purification.

For the structural study of apo state OR52_cs_, the chimeric construct of full-length OR52_cs_ (1–313)^23^ was designed to insert bRIL between A226 and L227 with a linker sequence derived from adenosine receptor A2a, as previously described^35^. For purification of active state OR52_cs_, full-length OR52_cs_ (1–313) was used. Each construct was subcloned into the pFastBac HT B vector with a Lucy signal sequence (MRPQILLLLALLTLGLA)^17^ and FLAG peptide at the N-terminus. Each construct also included an eGFP with 10xHis-tag, which was fused to the C-terminus of OR52_cs_ with a HRV 3C protease cleavage sequence.

For cAMP assay, CRE luciferase assay, and surface ELISA, the full-length OR52_cs_ construct was cloned into the pcDNA3.1 vector. The Lucy signal sequence and FLAG peptide sequence were added to the N-terminus. Mutant constructs were generated by site-directed mutagenesis. For BRET assay, eYFP was added at the C-terminus of OR52_cs_ with a GGGGS linker. Rluc was added to Gα for resonance partner (after L99 for Gα_s_, and I100 for Gα_olf_, respectively, with GGGGS linker back and forth).

### Sequence analysis

The amino acid sequences of human ORs were obtained from HORDE^46^ and pseudogenes were excluded for sequence analysis. The sequences of 388 intact human ORs or 26 members of OR52 family were aligned using MAFFT^47^ with L-INS-i strategy. MEGAX^48^ was used for phylogenetic tree generation with maximum-likelyhood method. The sequence conservation was visualized by WebLogo 3^49^.

### Purification of OR52_cs_-bRIL

OR52_cs_-bRIL was overexpressed in *Spodoptera frugiperda* (*Sf9*, Expression Systems, 94-00lF) cells using the Bac-to-Bac baculovirus expression system (Invitrogen). Cells were harvested 60 h after infection and lysed with a buffer containing 20 mM Tris-Cl (pH 8.0), 150 mM NaCl, 1 mM phenylmethylsulfonyl fluoride (PMSF), 175 µg mL^−1^ benzamidine, and 10 µM leupeptin using a dounce homogenizer. OR52_cs_-bRIL was extracted from the membrane fraction with solubilization buffer containing 20 mM Tris-Cl (pH 8.0), 150 mM NaCl, 1% (w/v) lauryl maltose neopentyl glycol (LMNG, Anatrace) and 0.1% (w/v) cholesteryl hemisuccinate (CHS, Sigma-Aldrich), 1 mM PMSF, 175 µg mL^−1^ benzamidine, and 10 µM leupeptin. After centrifugation, the supernatant was loaded onto a Ni-NTA column (Qiagen) pre-equilibrated with wash buffer consisting of 20 mM Tris-Cl (pH 8.0), 10 mM imidazole, 150 mM NaCl, 0.005% (w/v) LMNG, and 0.0005% (w/v) CHS. After washing the column with wash buffer, OR52_cs_ was eluted with 250 mM imidazole buffer. The eluted sample was loaded onto anti-FLAG affinity (M1) column (Sigma-Aldrich) pre-equilibrated with LMNG buffer (20 mM Tris-Cl (pH 8.0), NaCl 150 mM, 0.005% (w/v) LMNG and 0.0005% (w/v) CHS) supplemented with 2 mM CaCl_2_. OR52_cs_-bRIL was eluted with LMNG buffer containing 0.1 mg ml^−1^ FLAG peptide and 4 mM EDTA, and further purified with size exclusion chromatography (SEC) using a Superdex 200 10/300 column (Cytiva), which was pre-equilibrated with LMNG buffer. The peak fractions were collected and used to form the complex with bRIL-specific Fab.

### Purification of bRIL-specific Fab

Genes encoding the bRIL-specific Fab VH and VL domains^35^ were subcloned into the pFastBac Dual vector and Fab was expressed in BTI-Tn-5B1-4 (High Five, Expression Systems, 94-002 F) cells using the Bac-to-Bac expression system. Cells were harvested 72 h after infection, and the supernatant was collected and loaded onto a Ni-NTA resin pre-equilibrated with wash buffer containing 20 mM HEPES (pH 7.5), 200 mM NaCl, and 10 mM imidazole. After washing the column with wash buffer, Fab was eluted with 250 mM imidazole buffer and subsequently loaded onto a HiLoad 26/200 Superdex 200 column (Cytiva) for further purification.

### Purification of OR52_cs_-bRIL–Fab complex

Purified OR52_cs_-bRIL and bRIL-specific Fab were mixed at a molar ratio of 1:1.2 and incubated on ice for 30 min. The complex was purified with SEC using a Superdex 200 10/300 column (Cytiva) which was pre-equilibrated with 20 mM Tris-Cl (pH 8.0), 150 mM NaCl, 0.0025% (w/v) LMNG and 0.00025% (w/v) CHS to remove excess Fab. The peak fractions were concentrated to 9 mg ml^−1^ and used to prepare cryo-EM grids.

### Purification of the Nb35

Nanobody-35(Nb35) with a 6xHis-tag at its C-terminus was expressed in *Escherichia coli* (*E. coli*) Rosetta (DE3, Novagen) cells and purified as previously described^50^. Briefly, Nb35 was purified with Ni-NTA column, followed by SEC with Superdex 200 10/300 column (Cytiva). The peak fractions were collected and stored at 4 °C until use.

### Purification of OCA–OR52_cs_–G_s_–Nb35 complex

For purification of the OR52_cs_–G_s_–Nb35 complex, OR52_cs_, human Gα_s_, human Gβ_1_, and human Gγ_2_ were co-expressed in *Sf9* insect cells using a Bac-to-Bac expression system. Cells were harvested 60 h after infection and lysed with a buffer containing 20 mM Tris-Cl (pH 8.0), 150 mM NaCl, 1 mM PMSF, benzamidine, and leupeptin. After centrifugation, the pellet was resuspended in a buffer containing 20 mM Tris-Cl (pH 8.0), 150 mM NaCl, 10 µg ml^−1^ Nb35, 25 mU ml^−1^ apyrase, 2 mM octanoic acid (OCA, Sigma-Aldrich), 1 mM PMSF, benzamidine, and leupeptin, to form the complex in the membrane, and the membrane was solubilized with 1% (w/v) LMNG (Anatrace) and 0.1% (w/v) CHS at 4 °C for 2 h. After centrifugation at 21,671 × *g* for 15 min, the supernatant was loaded onto a Ni-NTA column and the column was washed with LMNG buffer containing 10 mM imidazole and 2 mM OCA. The complex was eluted with 200 mM imidazole and loaded onto an M1 column pre-equilibrated with LMNG buffer containing 2 mM CaCl_2_ and 2 mM OCA. The resin was washed with the same buffer, and the protein complex was eluted with LMNG buffer supplemented with 0.1 mg ml^−1^ FLAG peptide, 3 mM EDTA, and 2 mM OCA. The protein complex was confirmed by SDS-PAGE gel and concentrated for cryo-EM grid preparation.

### Purification of olfactory Gα subunit (Gα_olf_)

To purify Gα_olf_, 6xHis-Gα_olf_ was co-expressed with GST-Ric8b in *Sf9* cells. Cells were harvested 48 h after infection and lysed with lysis buffer (20 mM Tris-Cl, (pH 8.5), 100 mM NaCl, 1 mM EDTA, and protease inhibitors). After centrifugation (21,671 × *g*, 20 min, 4 °C), the supernatant was loaded onto a Ni-NTA column. After column washing, Gα_olf_ and Ric8b complexes were eluted with elution buffer (20 mM Tris-Cl (pH 8.5), 150 mM NaCl, 300 mM imidazole, 0.1 mM Tris (2-carboxyethyl) phosphine hydrochloride (TCEP)). The eluted fractions were collected and further purified using the Hitrap Q anion-exchange column (Cytiva). The peak fractions were pooled and concentrated, and GTPγS and MgCl_2_ were added to separate Gα_olf_ from Ric8b. After 1 h incubation at room temperature, the reaction mixture was loaded onto Hitrap Q and Gα_olf_ was separately eluted from the column. Purified Gα_olf_ was concentrated to 8 mg ml^−1^ and used for crystallization.

### Crystallization, data collection, and structure determination of Gα_olf_

GTPγS-bound Gα_olf_ was crystallized by hanging drop vapor diffusion at 22 °C with reservoir solution consisting of 0.2 M MgCl_2_ and 18% PEG 3350. Crystals were flash-cooled in liquid nitrogen using 20% ethylene glycol as a cryoprotectant.

Diffraction data were collected at 100 K on beamline 5 C at the Pohang Accelerator Laboratory (PAL, Korea) and processed with the XDS package^51^. The structure of Gα_olf_ was solved by molecular replacement with Phaser^52^, using the GTPγS-bound Gα_s_ structure (PDB:1AZT) as a search model. Iterative cycles of manual rebuilding with Coot^53^ and refinement with PHENIX^54^ were performed to obtain the final model, which was validated with Molprobity^55^. The final model was deposited in the PDB with PDB code 8HTG.

### Cryo-EM grid preparation and data collection

3.5 µl of the purified OR52_cs_-bRIL–Fab and OCA–OR52_cs_–G_s_–Nb35 complexes (9 mg ml^−1^ and 5 mg ml^−1^, respectively) were applied to the glow-discharged 300 mesh holey carbon grid (Quantifoil R1.2/1.3) (SPI) pre-coated with poly-L-lysine (Sigma-Aldrich) and 300 mesh R1.2/1.3 UltraAuFoil grid (SPI), respectively. Each grid was blotted for 3 s with a blot force of 5 at 4 °C, 100% humidity, and plunge-frozen in liquid ethane using a Vitrobot Mark IV (Thermo Fisher Scientific). The grids were initially screened with FEI Glacios (Thermo Fisher Scientific) equipped with a Falcon 4 detector (Thermo Fisher Scientific) at the Center for Macromolecular and Cell Imaging at Seoul National University (SNU CMCI, Korea).

Images of the OR52_cs_-bRIL–Fab complex were collected using a Titan Krios G4 (Thermo Fisher Scientific) at the Institute of Basic Science (IBS, Korea), equipped with a K3 BioQuantum (Gatan) at a magnification of 105,000X with a calibrated pixel size of 0.848 Å. Movies were collected with a total dose of 68.5 e^−^ A^−2^ and 57 frames per micrograph with a defocus ranging from −0.7 to −1.9 μm.

Data for the OCA–OR52_cs_–G_s_–Nb35 complex were collected using a Titan Krios G4 at the Institute of Membrane Proteins (IMP, Korea), equipped with a K3 BioQuantum at a magnification of 105,000× with a calibrated pixel size of 0.851 Å. Movies were collected at a total dose of 60.2 e^−^ A^−2^ and 50 frames per micrograph with a defocus ranging from −0.8 to −2.0 μm.

### Cryo-EM data processing and 3D reconstruction

Image stacks were subjected to beam-induced motion correction using the patch motion correction, and the contrast transfer function (CTF) parameters for each non-dose-weighted micrograph were determined using patch CTF estimation. Data processing was mostly performed using cryoSPARC^56^.

For the OR52_cs_-bRIL–Fab complex, 21,345 movies were used for data processing using cryoSPARC v4.2.0. Particles were initially selected using a Blob picker. Templates were generated by performing several rounds of two-dimensional (2D) classification and template selection. The final selected particles were used for Topaz training^57^, and the extracted particles were used to reconstruct the 3D volume. Multiple rounds of Ab-initio reconstruction and heterogeneous refinement were performed to select suitable particles. Non-uniform refinement^58^ from the selected 440,134 particles resulted in a Fab-focused map with a global resolution of 3.44 Å. A mask covering OR52_cs_ was generated using the Chimera built-in Segger tool and masked 3D classification was performed to isolate the particles that could reconstruct intact TMs of OR52_cs_. The selected 173,732 particles were beam-tilt corrected by global CTF refinement and reached to 3.66 Å resolution map (volume-1) with intact TMs of OR52_cs_. Local refinement with an OR52_cs_-focused mask produced a map with a resolution of 3.74 Å. The additional 3D classification was performed to discard bad particles, and the final 142,861 particles went through local refinement which produced a OR52_cs_-focused map (volume-2) with a resolution of 3.39 Å. The resolution of the final map was also estimated by RELION v3.1.1 with each half maps^59^, which results in resolution of 3.6 Å. The output volume-2 was used for the structure determination of OR52_cs_. Because volume-2 was focused on OR52_cs_ and not sufficient for model building of bRIL, volume-1 was used for structure determination of linker and bRIL. The detailed description of the cryo-EM data collection parameters is provided in Supplementary Table 2.

For the OCA–OR52_cs_–G_s_–Nb35 complex, 4798 movies were used for data processing by cryoSPARC v3.2.2. Particles were selected by 2D classification, and selected particles were used for the Topaz training. 493,826 particles from the Topaz extract were separated by several rounds of 2D classification and heterogeneous refinement, and 74,356 particles were further refined by non-uniform refinement. Because the map has a preferred orientation issue, new templates were created for template picking to excavate the top- and bottom-view particles. Duplicate particles were removed and remaining particles were classified using heterogeneous refinement. The final selected particle sets were further subjected to beam-induced motion correction using a local motion correction tool and refined by non-uniform refinement to a global resolution of 2.97 Å. Masks covering OR52_cs_ or G_s_–Nb35 were generated using Chimera built-in Segger tool. Local refinement was performed to refine the OR52_cs_ and G_s_–Nb35 parts. Finally, 126,896 particles were used to yield a 3D map with a local resolution of 3.09 Å for OR52_cs_ and 2.84 Å for G_s_–Nb35. These maps were combined using the Vop maximum tool built-in Chimera, and the resulting map was used for model building. The detailed description of the cryo-EM data collection parameters is provided in Supplementary Table 2.

### Model building and refinement

For the OR52_cs_-bRIL–Fab complex, the protein sequence of OR52_cs_ was submitted as the input protein sequence of AlphaFold2.ipynb^41^, and one of the created models was used as the initial model. For the OCA–OR52_cs_–G_s_–Nb35 complex, the same AlphaFold model was used as the initial model. Models of the G_s_ heterotrimer and Nb35 were derived from the crystal structure of the active β_2_AR–G_s_ protein complex (PDB:3SN6). Model building and refinement were performed by iterative cycles of refinement with PHENIX^60^ and manual rebuilding with Coot^53^. For the OR52_cs_-bRIL–Fab complex, the quality of OR52_cs_ model was assessed by Q-score calculating using MapQ software^61^. To validate the current OCA conformation and pose in our structure, we checked the conformational stability of OCA and the agreement of the OCA structure with the density by running the Rosetta relax application (dual-space relax)^62^. The criteria for conformational instability of the ligand can be assessed by high RMSD values (more than 2 Å) during the simulations. Relax runs were repeated ten times with and without the electron density information. In both types of simulations, a strong convergence towards the pre-relaxed original conformation (RMSD < 0.8 Å) was observed from the sampled ligand conformations, supporting that our original conformation is stable and agrees well with the electron density. Refinement statistics are presented in Supplementary Table. 2. In the final model of the active structure, the N-terminal seven residues (1–7) and the C-terminal three residues (311-313) of OR52_cs_, and α-helical region (47–193) as well as the N-terminal nine residues (1–9) and some loop regions (235–249, 281–294, and 308–319) of Gα_s_ were not modeled due to poor map quality. In the case of the apo structure, the N-terminal 11 residues (1-10, 18), ICL1 (53-56), part of ECL2 (169–173, 190–193), helix 8, and the C-tail (295–313) were not modeled.

### BRET assay

All signaling assays were performed in Hana3A cells^22^, which were kindly provided by Dr. Matsunami. Cells were seeded on 6-well clear plate (SPL) and co-transfected with OR52_cs_-eYFP, Gα_s_-Rluc or Gα_olf_-Rluc, Gβ_1_ and Gγ_2_ at a 5:1:1:1 ratio. 48 h after infection, cells were collected and resuspended with PBS. 500 μM of OCA was treated to the cells, and cells were transferred to 96-well white microplate (SPL). Coelenterazine H (Nanolight) was added to each well at a final concentration of 10 μM and luminescence was measured using a Tristar 2 LB 942 multimode reader (Berthold). Data were analyzed using GraphPad Prism 9.4.1.

### cAMP signaling assay

Hana3A cells were seeded on 96-well white microplates and transfected with OR52_cs_ or the corresponding mutants and cAMP sensor (Promega). The medium was exchanged with CO_2_-independent medium (Gibco) supplemented with 0.375 mg ml^−1^ D-luciferin (Nanolight) 48 h after infection, and cells were incubated in the dark at 20 °C for 2 h. Basal luminescence was detected using a Tristar 2 LB 942 multimode reader (Berthold) until it reached a plateau. Subsequently, various concentrations of odorants and forskolin (Sigma-Aldrich) were added to each well, and luminescence was measured again. Data were analyzed and plotted using GraphPad Prism 9.4.1.

### CRE assay

Hana3A cells were seeded on 96-well white microplates (SPL) and transfected with plasmids encoding CRE-luciferase, OR52_cs_ or R265^6.59^A mutant, Renilla luciferase, RTP1S, and Ric8b at a 5:1:1:1:1:1 ratio 24 h after seeding. 48 h after infection, cells were treated with odorants and incubated at 37 °C for 4 h. Luminescence was measured using a Tristar 2 LB 942 multimode reader (Berthold). All values were divided by the Renilla luciferase activity to normalize the effect of cell confluence. All data were analyzed using GraphPad Prism 9.4.1.

### ELISA-based surface expression assay

Hana3A cells were seeded on 96-well clear microplates (NEST) and transfected with plasmids encoding OR52_cs_ or its corresponding mutants. All the constructs contained a FLAG tag at the N-terminus. Cells were treated with 4% paraformaldehyde for fixation 48 h after infection and washed with PBS. After 2 h of incubation with blocking solution (5% bovine serum albumin (BSA, Bovogen) in PBS), rabbit anti-FLAG antibody (Cell Signaling Technology, D6W5B, 1:1,000 dilution) was treated at 4 °C overnight. Cells were washed extensively with PBS and treated with goat anti-rabbit horseradish peroxidase (HRP)-conjugated antibody (Enzo Life Sciences, ADI-SAB-300-J, 1:1000 dilution). After 2 h of incubation, the TMB solution (Thermo Fisher Scientific) was added to each well, and when a blue color appeared, 1 M HCl was added to quench the reaction. The absorbance was measured at 450 nm using a FlexStation3 microplate reader (Molecular Devices). For normalization, Janus green solution (0.2% (w/v), TCL) was added to the cells and mixed with 0.1 M HCl. The absorbance was measured at 595 nm. The normalized expression level of the receptor at the cell surface was calculated as the ratio between the absorbance at 450 nm and 595 nm (A_450_/A_595_).

### All-atom MD simulations of apo and OCA-bound states of OR52_cs_

Three model systems were prepared for all-atom MD simulation: OCA–OR52_cs_–G_s_ (139 × 139 × 171 Å^3^), OCA–OR52_cs_ (91 × 91 × 113 Å^3^), and apo OR52_cs_ (90 × 90 × 116 Å^3^), with the total numbers of atoms (and water molecules) of 308,264 (221,229), 85,555 (17,823), and 87,389 (18,441), respectively (Supplementary Table 6). The receptor was embedded into a model membrane composed of 1-palmitoyl-2-oleoyl-sn-glycero-3-phosphocholine (POPC) and cholesterol (4:1). For the G protein, three lipidations were introduced into the Gα_s_ and Gγ proteins, i.e., N-myristoylation (Gly2 of Gα_s_), S-palmitoylation (Cys3 of Gα_s_), and S-geranylgeranylation (C68 of Gγ). We used the CHARMM-GUI PDB Reader & Manipulator, Ligand Reader & Modeler, Membrane Builder, and Input Generator^63–68^.

The CHARMM36(m) force field^69,70^ was utilized for lipids and proteins, and CGenFF^71^ was used for the OCA ligand. For solvation, 0.15 M KCl was included in the TIP3P water model^72,73^. The van der Waals interactions were switched off smoothly between 10 and 12 Å by a force-based switching function^74^, and the particle-mesh Eward method^75^ with a mesh size of ~1 Å was used to calculate the long-range electrostatic interactions. The SHAKE algorithm^76^ was used for constraining bond lengths including hydrogen atoms, and the temperature and pressure were set to 303.15 K and 1 bar, respectively, with Langevin dynamics with a friction coefficient of 1 ps^−1^ and Monte Carlo barostat^77^. Following the CHARMM-GUI six-step equilibration procedure^78^, NVT simulations (constant particle number, volume, and temperature) with positional and dihedral restraints were performed for equilibration by gradually alleviating the force constant, and then NPT (constant particle number, pressure, and temperature) simulations were conducted for production run without restraints with 4 fs time-step using the hydrogen mass repartitioning method^79,80^. Periodic boundary condition was applied for all simulations, and we conducted five independent simulations for each system for effective sampling. Simulations were performed at least 1 μs for five replicas using OpenMM simulation package^81^.

Based on the OpenMM simulations, we utilized Anton2 to extend our investigation of the systems’ dynamics at a longer time scale up to 20 µs and 10 µs for OCA-OR52_cs_ and OCA-OR52_cs_-G_s_, respectively^82^. In Anton2 simulations, we used a time step of 2 fs and saved frames every 240 ps, where NPT ensemble with Nosé-Hoover method and Multigrator integrator were used for temperature and pressure coupling, respectively^83,84^. Although we conducted extensive simulations for these systems, the sampling may vary when reproduced.

### Docking and MD relaxation of different lengths of carboxylic acids

The OR52E5 receptor structure was predicted using AlphaFold2^41^ supported by the OCA–OR52_cs_–G_s_ structure as a template, and refined using Rosetta FastRelax^62^. To model the odorant–OR52E5 interaction, complex structures for various ligands ranging from hexanoate to dodecanoate were created by using Rosetta GALigandDock^85^. To estimate the stability of the docked structures, MD relaxations were carried out using pmeMD_cuda from AMBER20 in GPUs^86^. CHARMM-GUI was used for input generation in the same manner as that described in the previous section. POPE and POPC lipid bilayers were mixed at 1:1 and TIP3P water^72,73^ at 0.15 M KCl were employed. The Amber force field AMBER19SB, LIPID17, and GAFF2 were applied to the protein, lipids, and ligand. 200 ns production run was performed at 303 K. Changes in the receptor structure were analyzed using the CPPTRAJ utility in AMBER^87^. As a measure for the structural stability of each receptor–ligand complex, the RMSD of the binding pocket residues (H107, T110, F161, H183, A202, V205, F258, and R262) from the initial docked structure during the simulation trajectory was measured. Structural modeling of pentanoate–OR52L1 was conducted by using the same procedure as for OR52E5.

### Reporting summary

Further information on research design is available in the Nature Portfolio Reporting Summary linked to this article.

### Supplementary information

Supplementary Information

Peer Review File

Reporting Summary

### Source data

Source Data

## Data Availability

The coordinate and structure factor of GTPγS-bound Gα_olf_ were deposited in the Protein Data Bank under accession number of 8HTG. The coordinates of OR52_cs_ in the apo (with and without bRIL) and in complex with OCA, G_s_, and Nb35 were deposited in the Protein Data Bank under accession numbers of 8J46, 8WW7 and 8HTI, respectively. The cryo-EM density maps have been deposited in the Electron Microscopy Data Bank under accession numbers of EMD-35971 (globally refined cryo-EM density containing apo state OR52_cs_, bRIL, and bRIL-Fab), EMD-37336 (OR52_cs_-focused refined cryo-EM density of apo state OR52_cs_), EMD-35010, EMDB-35770 (composite (OR52_cs_-focused and G_s_-focused merged) cryo-EM density of OCA–OR52_cs_–G_s_–Nb35 complex), EMD-35772 (OR52_cs_-focused refined cryo-EM density of OCA–OR52_cs_–G_s_–Nb35 complex), and EMD-35773 (G_s_-focused refined cryo-EM density of OCA–OR52_cs_–G_s_–Nb35 complex). The initial and final configurations obtained from 1-µs all-atom MD simulations and extended simulations from Anton2 of all model systems are available at https://github.com/sek24/natcomm2023. Source data are provided with this paper.
