# Supplementary material for: Understanding the molecular mechanisms of odorant binding and activation of the human OR52 family
Source: Nat Commun. 2023 Dec 7;14:8105. doi: 10.1038/s41467-023-43983-9 (PMC10703812; doi:10.1038/s41467-023-43983-9)

# Understanding the molecular mechanisms of odorant binding and activation of the human OR52 family

Chulwon Choi†, Jungnam Bae†, Seonghan Kim, Seho Lee, Hyunook Kang, Jinuk Kim, Injin Bang, Kiheon Kim, Won-Ki Huh, Chaok Seok, Hahnbeom Park, Wonpil Im, Hee-Jung Choi*

Supplementary Figure 1-16
Supplementary Table 1-6
References
MD simulation checklist
Uncropped gel images

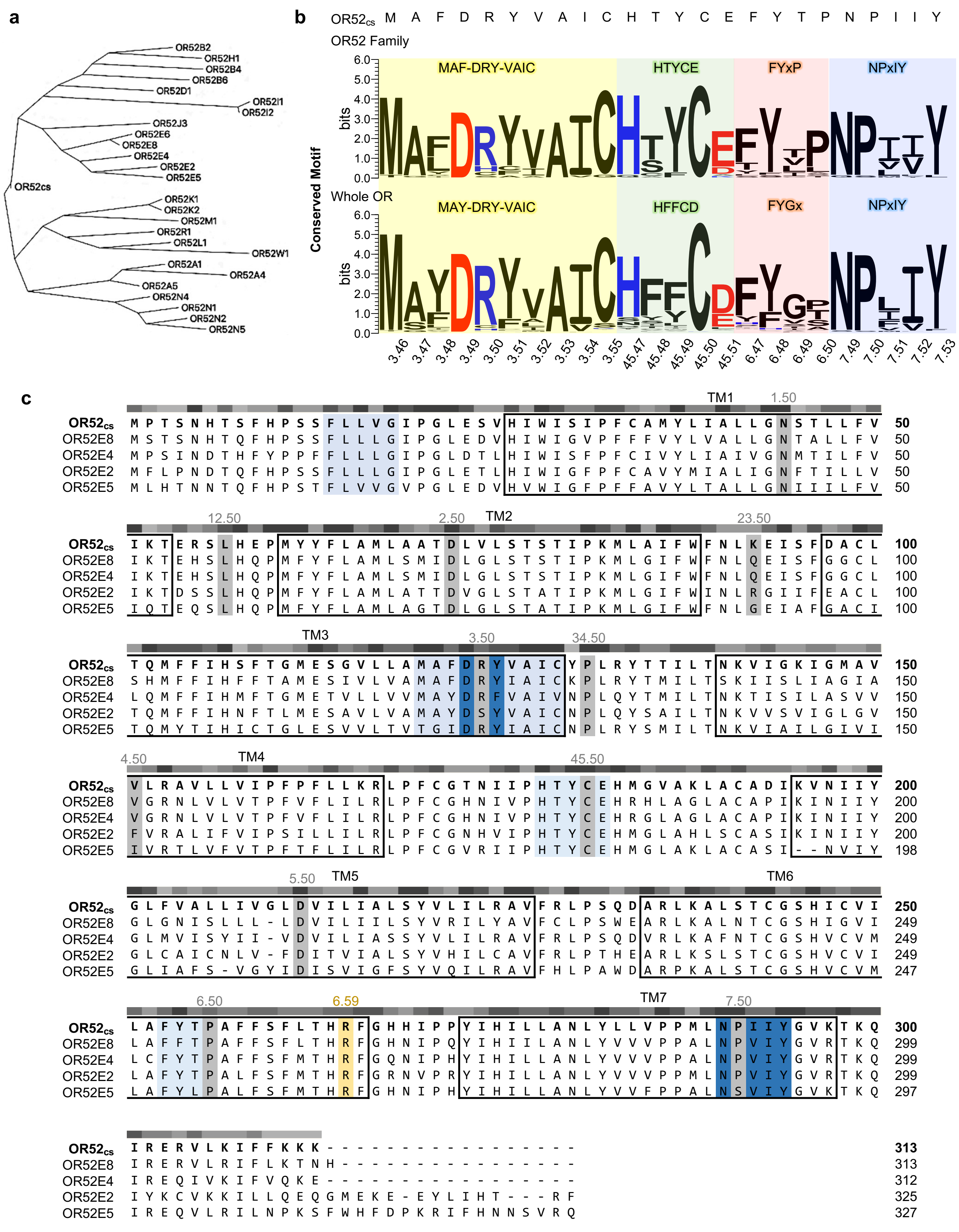


**Supplementary Fig. 1| OR52$_{cs}$ as representative of the human OR52 family by multiple sequence alignment. a** For OR52$_{cs}$ and each member of the human OR52 family, the phylogeny tree constructed by the maximum likelihood method is shown. **b** Among 26 human OR52 family members and whole 388 human ORs[1], the sequence conservation is displayed for OR-specific motifs. The graphical representation was depicted by WebLogo3[2]. **c** Sequence alignment of OR52$_{cs}$ with four native OR52 family members. OR52E8, OR52E4, OR52E2, and OR52E5 were selected for high sequence identity (> 65 %). OR-specific and class A GPCRs-conserved motifs were colored light blue and blue, respectively. Each TM is marked as a square and labeled. The most conserved position in class A GPCRs for each TM and loop are highlighted with a gray box and labeled with a generic number[3]. The conserved 6.59 position on TM6 was colored yellow. Above each position, the degree of conservation among the OR52 family is color-coded from gray (low) to black (high).

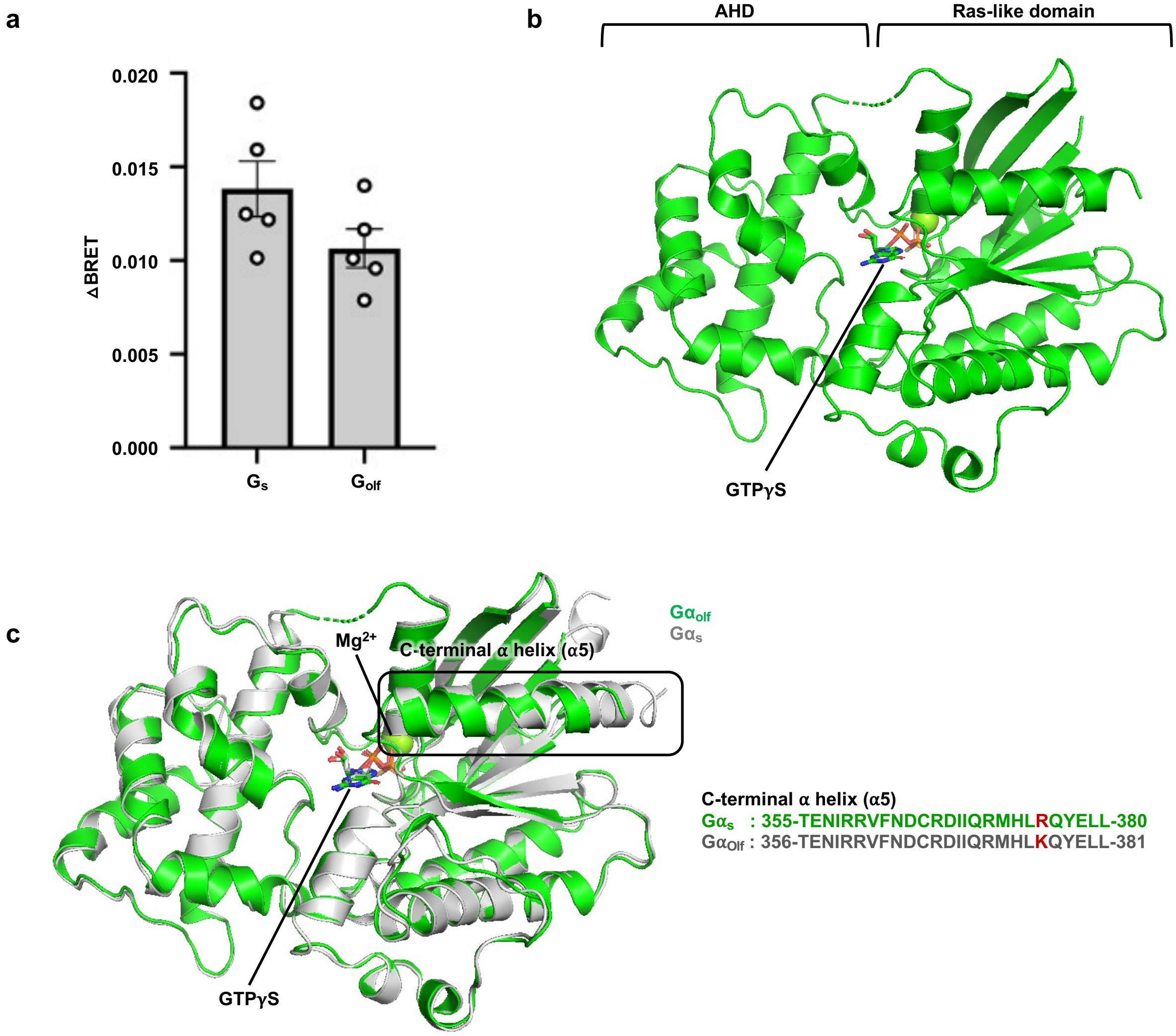


**Supplementary Fig. 2| Structural similarity between G$\alpha_s$ and G$\alpha_{olf}$. a** OCA-induced G protein recruitment to $OR52_{cs}$ was assessed with Bioluminescence Resonance Energy Transfer (BRET) assay. ΔBRET is calculated by the difference between 500 μM OCA- and vehicle-treated BRET signals. Bars and error bars indicate the mean and S.E.M. from n=5 independent experiments, respectively, and symbols indicate individual values. **b** The crystal structure of GTPγS-bound G$\alpha_{olf}$ is shown. Two domains of G$\alpha_{olf}$, AHD and Ras-like domain are indicated. **c** Structural alignment of G$\alpha_{olf}$ with a previously reported crystal structure of human G$\alpha_s$[4] (PDB ID 1AZT). The structure of G$\alpha_{olf}$ and G$\alpha_s$ are colored in green and white, respectively. Two structures showed a RMSD of 0.48 Å for 285 Cα atoms, and GTPγS is located at the same position. Box highlights the α5 helices of the two Gα structures.

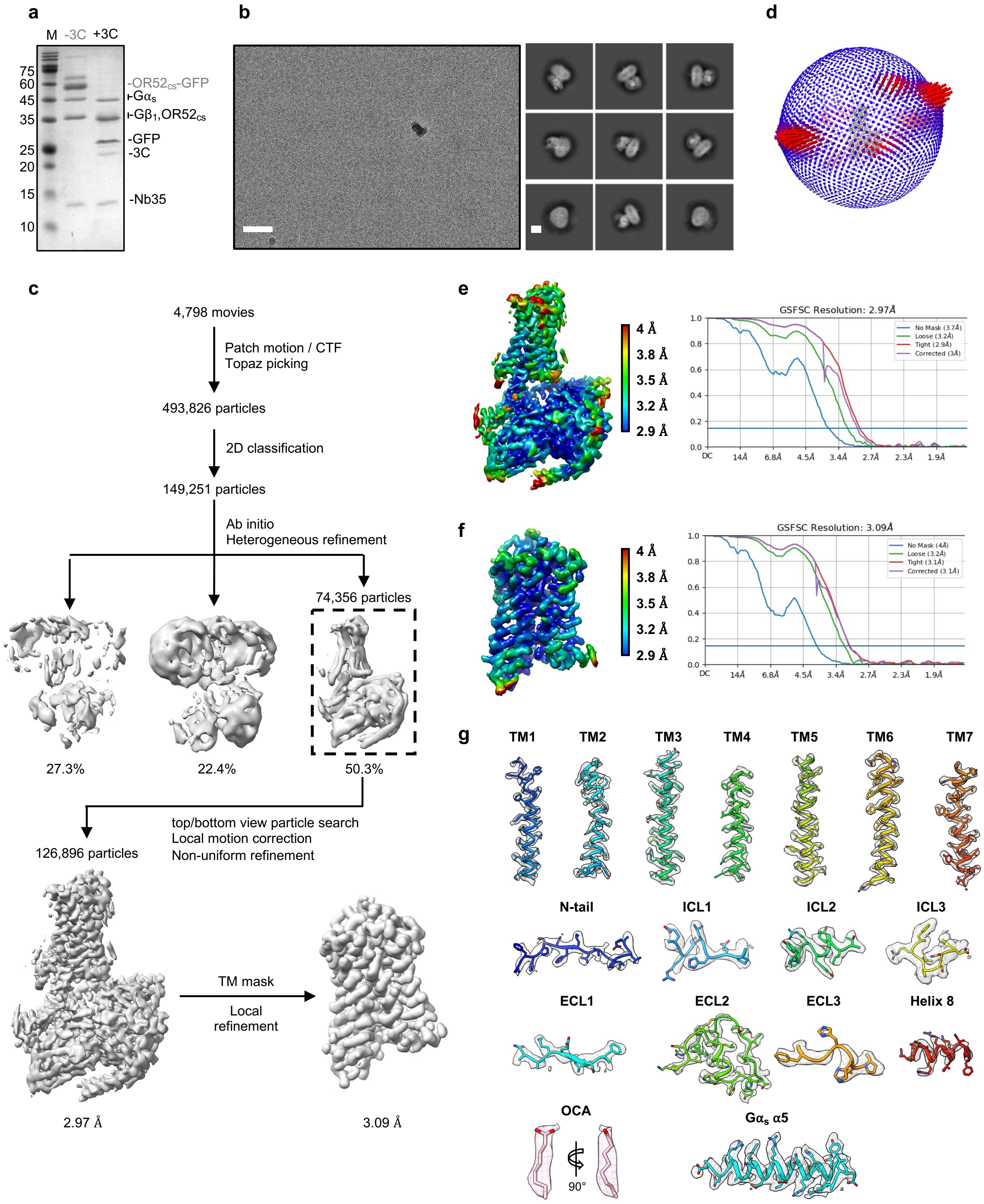


**Supplementary Fig. 3 Processing of cryo-EM data of the OCA–OR52$_{cs}$–G$_s$–Nb35 complex.** **a** SDS-PAGE of purified OCA–OR52$_{cs}$–G$_s$–Nb35 complex before and after HRV3C protease treatment. **b** Representative motion-corrected micrograph (scale bar, 50 nm) and 2D average classes (scale bar, 5 nm) of the complex are shown. **c** Flowchart of data processing using cryoSPARC v3.3.2[5, 6]. **d** The Euler angle distribution of particles used in final non-uniform refinement. **e-f** Cryo-EM maps colored by local resolution of Non-uniform refinement (**e**) and receptor-focused local refinement (**f**). **g** Density representation of TMs, N-tail, ECL1-3, ICL1-3, helix 8, and Gα$_s$ α5 helix are shown.

**a**

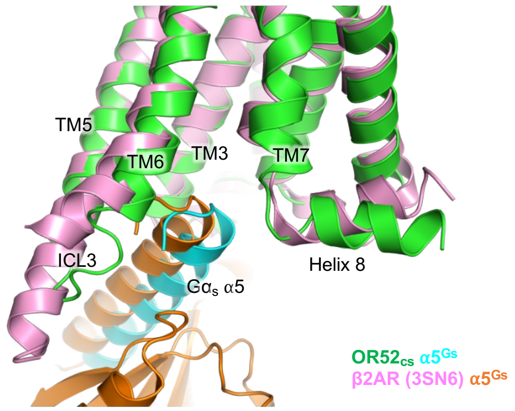


**b**

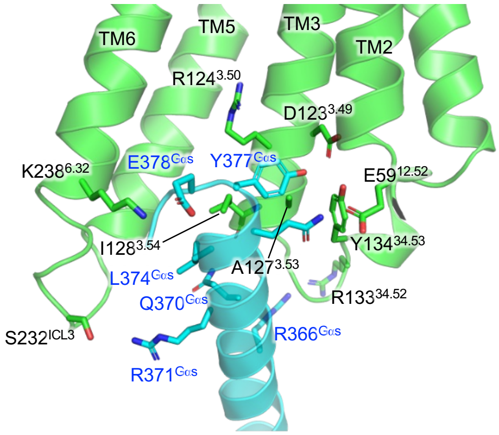


**c**

Sequence conservation

0% 100%

| Region | $OR52_{cs}$ | $G\alpha_s$ | Distance (Å) | Interaction character |
|---|---|---|---|---|
| ICL1 | E59 | Q376 | 2.6 | Hydrogen bond |
| TM2 | M61 | Q376 | 3.7 | van der Waals |
| | | Y377 | 3.8 | van der Waals |
| TM3 | D123 | Y377 | 2.4 | Hydrogen bond |
| | A127 | H373 | 3.2 | Hydrogen bond |
| | | Y377 | 3.6 | van der Waals |
| | I128 | Q370 | 3 | Hydrogen bond |
| | | L374 | 3.9 | van der Waals |
| ICL2 | C129 | R366 | 3.4 | Polar |
| | P131 | R366 | 3.8 | van der Waals |
| | | I369 | 3.7 | van der Waals |
| | L132 | F362 | 3.8 | van der Waals |
| | | R366 | 3.9 | van der Waals |
| | | I369 | 4 | van der Waals |
| | Y134 | Y377 | 3.8 | Polar |
| | T135 | Q35 | 3.4 | Polar |
| | | R38 | 3.6 | Polar |
| | T139 | Q35 | 3.8 | van der Waals |
| TM5 | I223 | L379 | 3.9 | van der Waals |
| | V227 | L374 | 3.9 | van der Waals |
| | L230 | L374 | 3.8 | van der Waals |
| ICL3 | S232 | R371 | 3.8 | Polar |
| | A235 | L380 | 3.6 | van der Waals |
| H8 | K297 | E378 | 2.9 | Hydrogen bond |

**d**

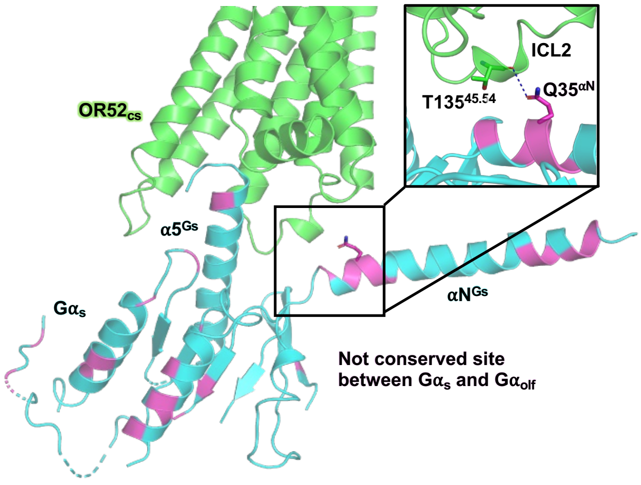


**αN (1–39)**

Q35αN

```
Gαs  MGCL-GNSK-TEDQRNEEKAQREANKKIEKQLQKDKQVYRA
Golf MGCLGGNSKTTEDQGVDEKERREANKKIEKQLQKERLAYKA
```

**α5 (356–380)**

```
Gαs  NIRRVFNDCRDIIQRMHLRQYELL
Golf NIRRVFNDCRDIIQRMHLKQYELL
```

**Supplementary Fig. 4| G protein binding interface of $OR52_{cs}$.** **a** Comparison of $G\alpha_s$ binding site between $OR52_{cs}$ and β2AR (PDB: 3SN6). **b** Interaction interface of $OR52_{cs}$ and α5 helix of $G\alpha_s$. Residues that participate in the interaction are shown as sticks. **c** Interactions between $OR52_{cs}$ and $G\alpha_s$, determined by the PyMol program (Molecular Graphics System v2.5.1, Schrödinger) are summarized. For each interaction, distance (Å) and character are displayed. For each residue of $OR52_{cs}$, the degree of conservation among whole 388 human ORs is color-coded. **d** Residues that are not conserved in $G\alpha_{olf}$ are highlighted in magenta in the structure of $G\alpha_s$. $Q35^{\alpha N}$ of $G\alpha_s$ is replaced with $L37^{\alpha N}$ in $G\alpha_{olf}$. For αN and α5 helices, sequence alignment between $G\alpha_s$ and $G\alpha_{olf}$ is shown at the bottom. Residues that differ between $G\alpha_{olf}$ and $G\alpha_s$ are colored red. All the $G\alpha_s$ residues that interact with $OR52_{cs}$, except for $Q35^{\alpha N}$ are conserved in $G\alpha_{olf}$. $Q35^{\alpha N}$ is replaced with $L37^{\alpha N}$ in $G\alpha_{olf}$, which will lose polar contact with the carbonyl group of $T135^{34.54}$.

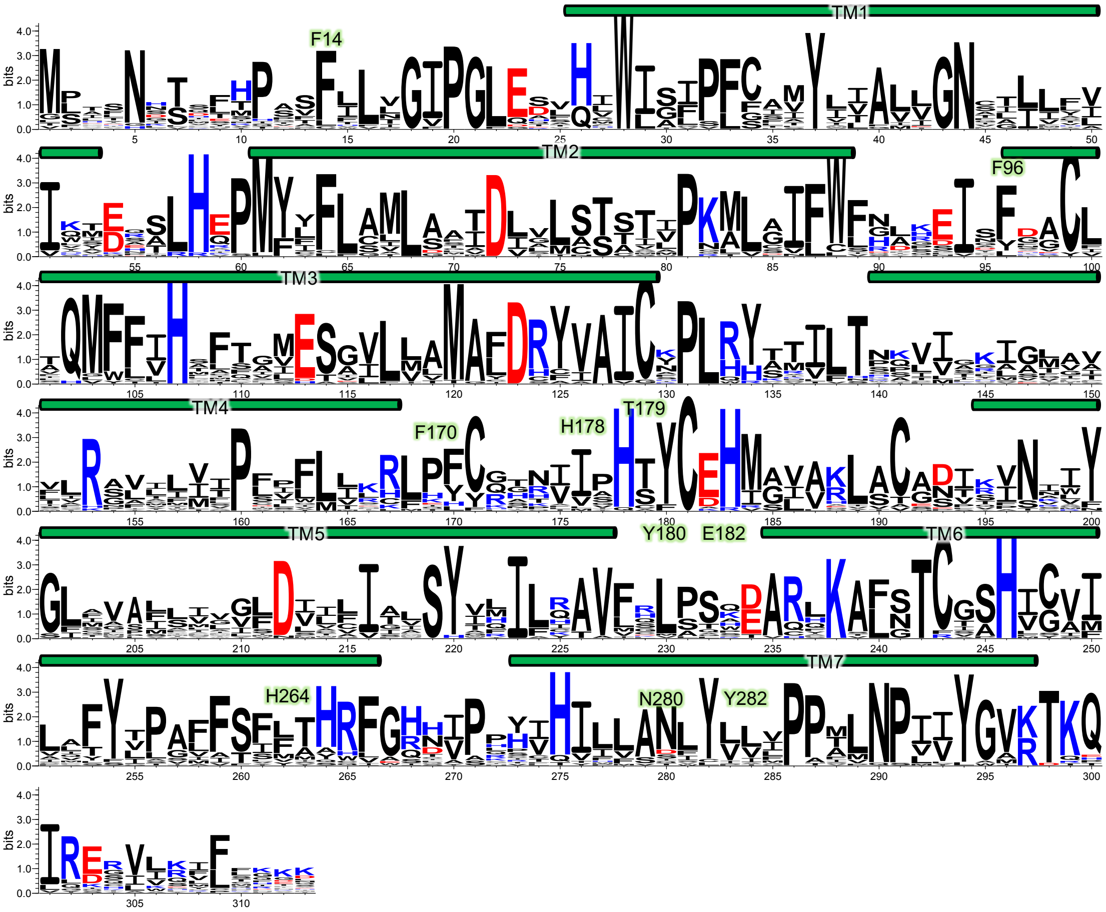


**Supplementary Fig. 5| Sequence conservation of the human OR52 family.** Sequence conservation of 26 human OR52 family members are shown. The position of each TM for $OR52_{cs}$ is shown. For each position, the residue number corresponds to that of $OR52_{cs}$. Conserved residues contributing to structural stability mentioned in **Fig. 2b-d** are marked with green text.

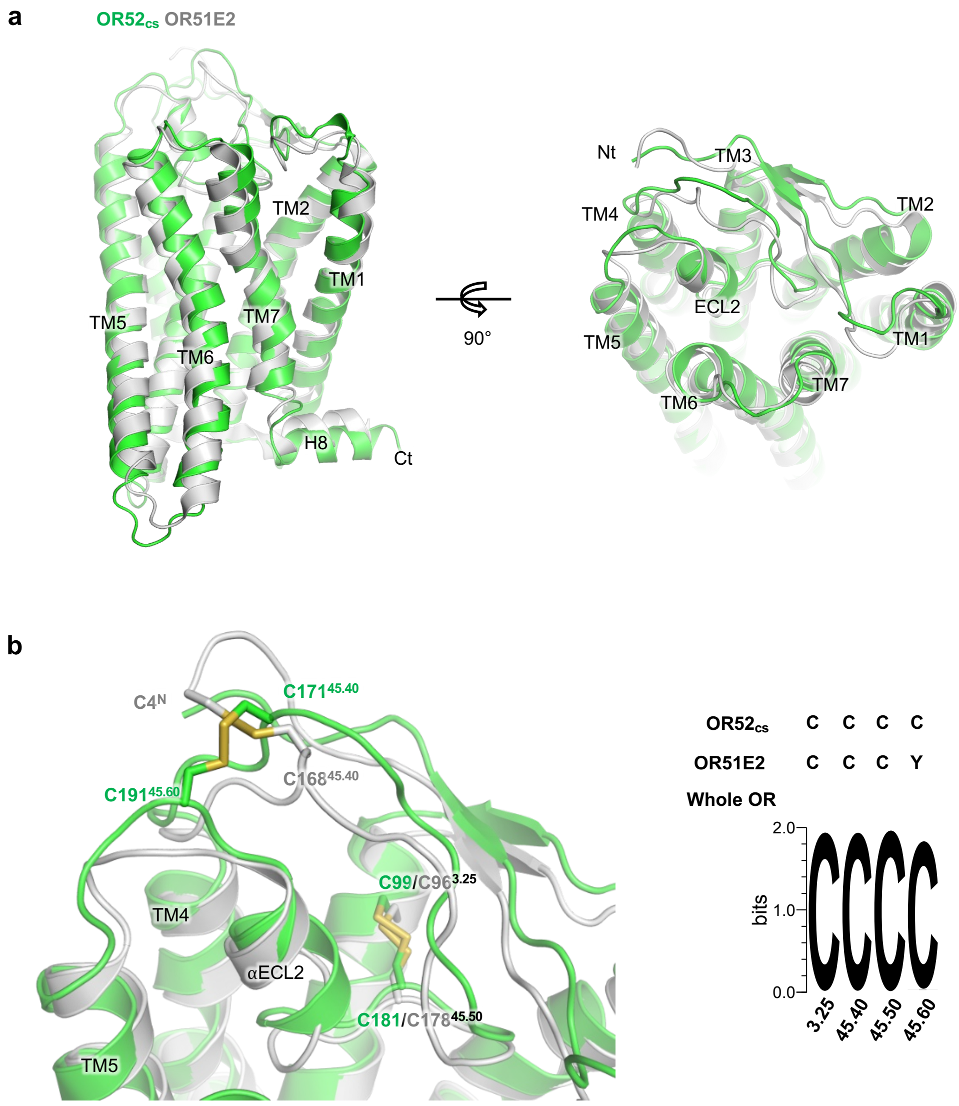


**Supplementary Fig. 6| Structural comparison of $OR52_{cs}$ and OR51E2. a** Active state $OR52_{cs}$ structure was aligned to OR51E2 (PDB:8F76) with a RMSD of 1.5 Å for 289 Cα atoms. **b** One of disulfide bonds are not conserved between the two structures. In OR51E2, highly conserved $C^{45.60}$ is replaced with Y.

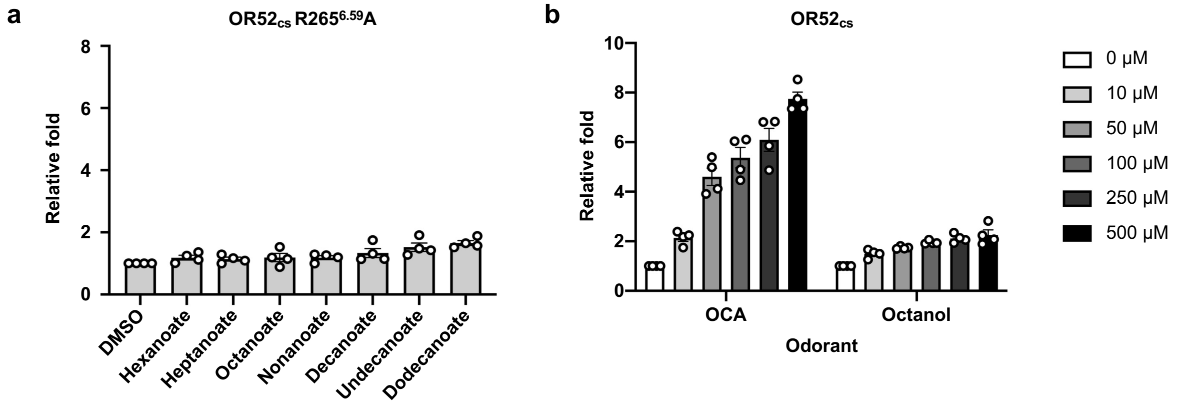


**Supplementary Fig. 7| CRE assays of R265$^{6.59}$A mutant and OR52$_{cs}$ with octanol. a** The activity of the OR52$_{cs}$ R265$^{6.59}$A mutant in response to the treatment of carboxylic acid odorants was assessed with CRE luciferase assay. Different lengths of carboxylic acids were treated at a final concentration of 100 μM. DMSO was used as negative control. Bars and error bars indicate the mean and S.E.M. of 4 independent experiments, respectively, and symbols indicate individual values. The relative fold was calculated by dividing the luciferase activity of each odorant with that of DMSO. Assays were done with Hana3A cell line and data were analyzed by GraphPad Prism 9.4.1. **b** The activity of OR52$_{cs}$ in response to OCA and octanol was assessed with CRE luciferase assay. Odorants were treated at five different concentrations as indicated on the right. Bars and error bars indicate the mean and S.E.M. of 4 independent experiments, respectively.

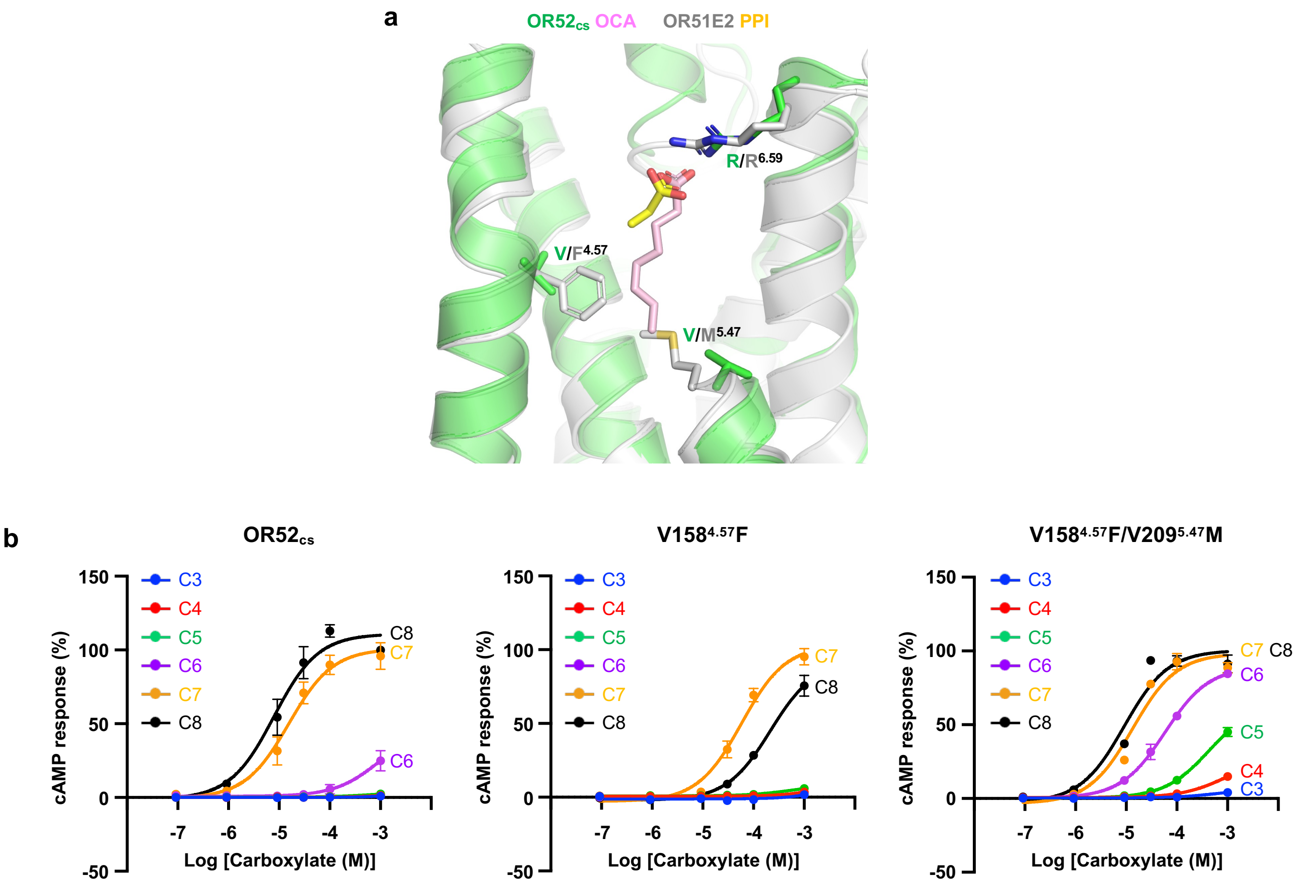


**Supplementary Fig. 8| Mutagenesis of odorant pocket residues of $OR52_{cs}$. a.** Structural alignment of $OR52_{cs}$ with OR51E2 is shown. OR51E2 is colored light-gray and PPI is shown as a yellow stick. Residues at positions 4.57, 5.47, and 6.59 are shown as sticks. **b.** Carboxylic acid odorants with various lengths were treated to $OR52_{cs}$ and two mutants ($V158^{4.57}F$ and $V158^{4.57}F/V209^{5.47}M$), and their cAMP response curves are shown. For each data, symbols and error bars indicate the mean and the standard error of the mean (S.E.M.) from n=3 independent experiments, respectively.

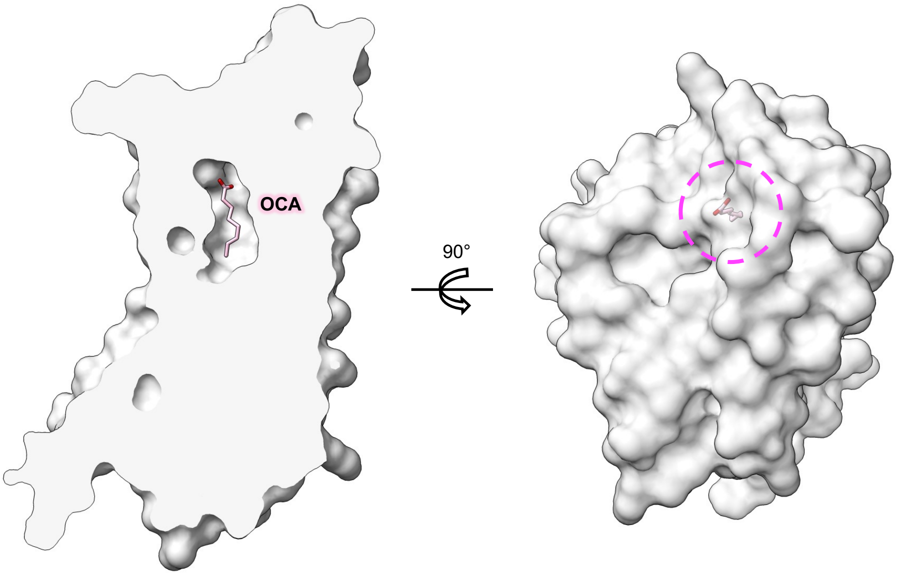


**Supplementary Fig. 9| The odorant binding pocket of OR52$_{cs}$.** The odorant binding pocket of OR52$_{cs}$ is shown using UCSF ChimeraX 1.6.1[7]. The surface model of the active OR52$_{cs}$ (light-gray) is shown and OCA (lightpink) is presented as sticks. Occluded odorant-binding pocket is indicated by a magenta dashed circle.

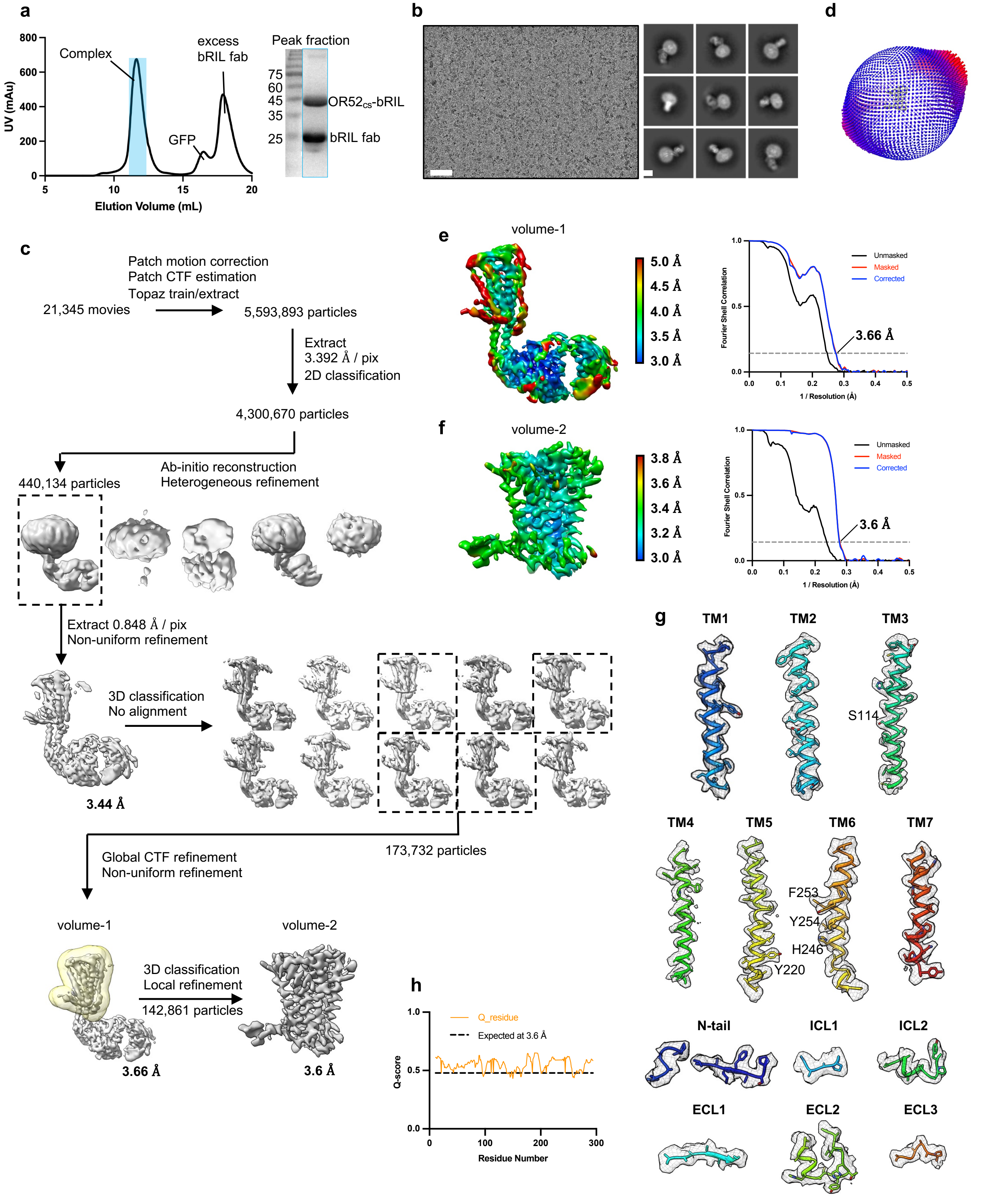


**Supplementary Fig. 10| Cryo-EM data analysis of OR52$_{cs}$-bRIL–Fab complex. a** SEC profile (left) and SDS-PAGE (right) of purified OR52$_{cs}$-bRIL–Fab complex. **b** Representative motion-corrected micrograph (scale bar, 50 nm) and 2D average classes (scale bar, 5 nm). **c** Flowchart of data processing using cryoSPARC v4.2.0. **d** The Euler angle distribution of final reconstructed local refinement map. **e-f** Cryo-EM maps colored by local resolution of Non-uniform refinement (**e**) and receptor-focused local refinement (**f**). FSC curve was calculated and exported from RELION v3.1.1[8]. **g** Density representation of TMs, N-tail, ECL1-3, ICL1-2 are shown. Key residues presented in **Fig. 5c, d** are labeled. **h** Q-scores estimated from final locally-refined map and model using MapQ v1.9.9 are plotted with expected Q-score value at 3.6 Å resolution[9].

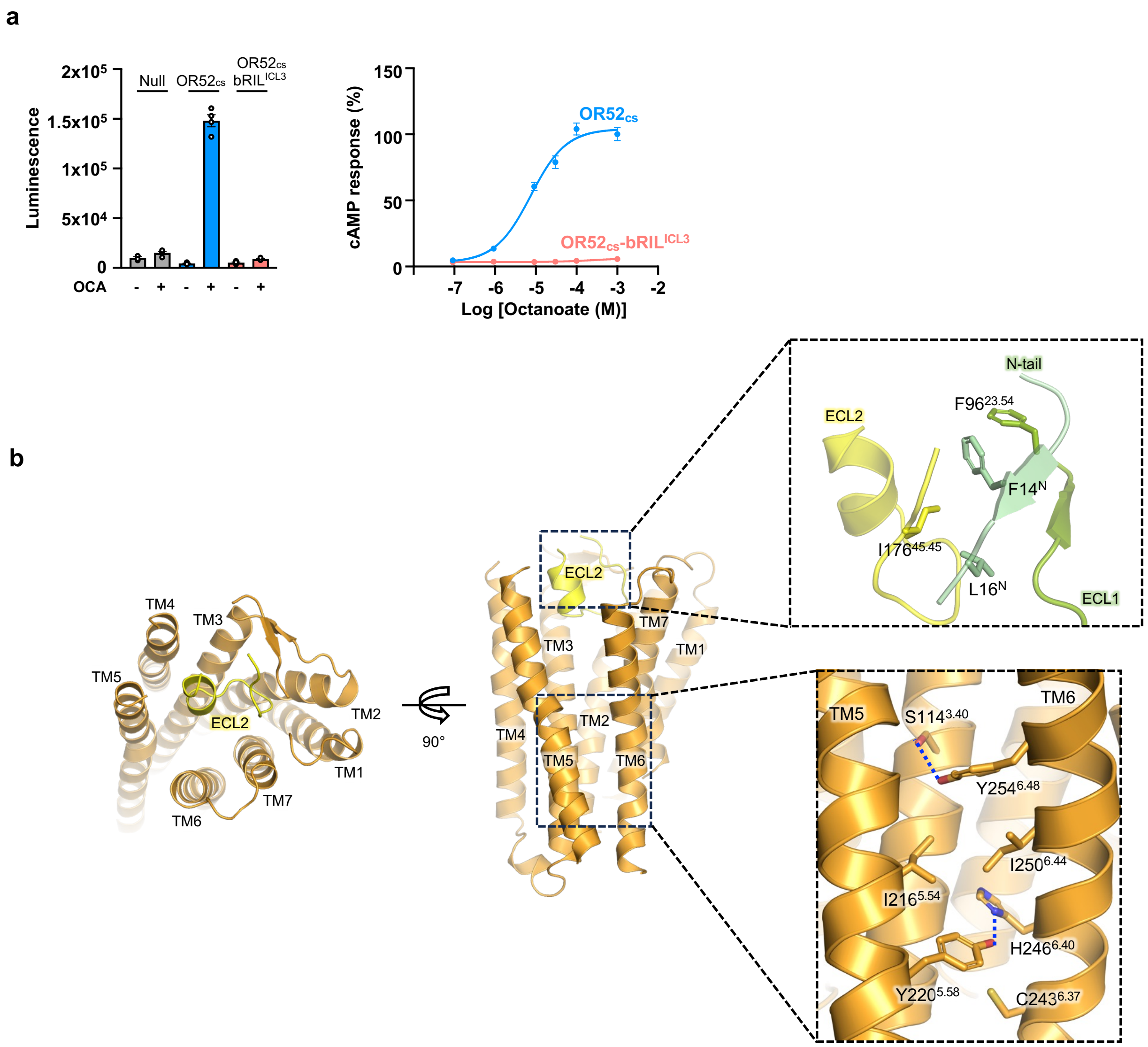


**Supplementary Fig. 11| Structural feature of ECL2 in apo state OR52$_{cs}$. a** Basal and odorant induced activities of OR52$_{cs}$ and OR52$_{cs}$-bRIL$^{ICL3}$ are assessed by cAMP responses upon DMSO or 1 mM OCA treatment (left). Dose-dependent cAMP response of each construct is presented (right). For each data, error bars indicate the standard error of the mean (S.E.M.) from n=4 independent experiments, respectively, and symbols indicate individual values. **b** The interactions that stabilize the apo structure near ECL2 and the FYxP motif are shown. Polar interactions are represented as blue dashed lines. The same color codes as in **Fig. 2b** are used for ECL2, N-tail, and ECL1.

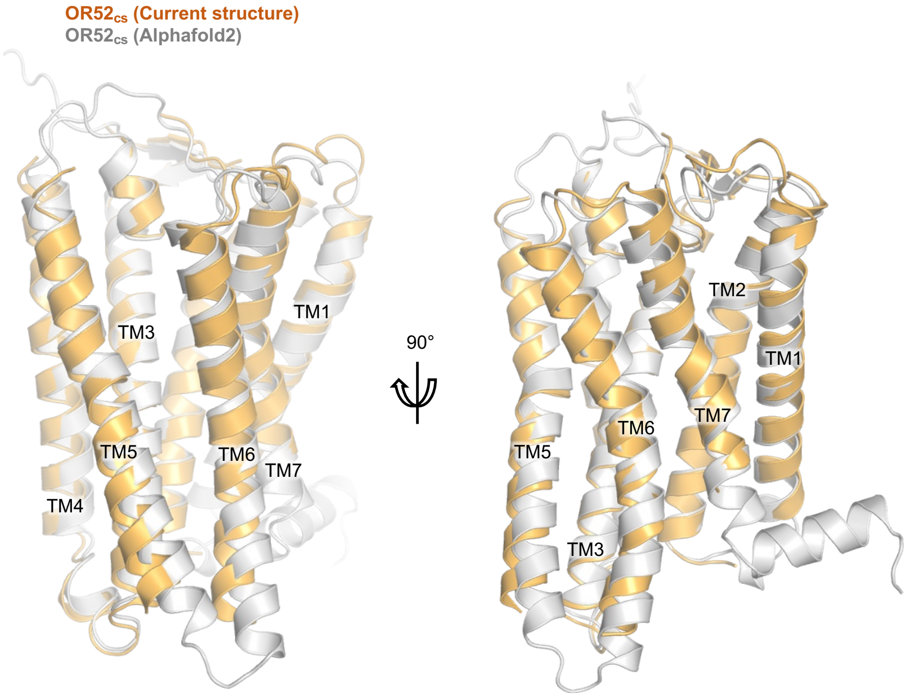


**Supplementary Fig. 12| Alphafold2−generated model of the apo state OR52$_{cs}$.** Alphafold2 predicted model was aligned with our apo state OR52$_{cs}$ structure, with a RMSD of 1.1 Å for 242 Cα atoms.

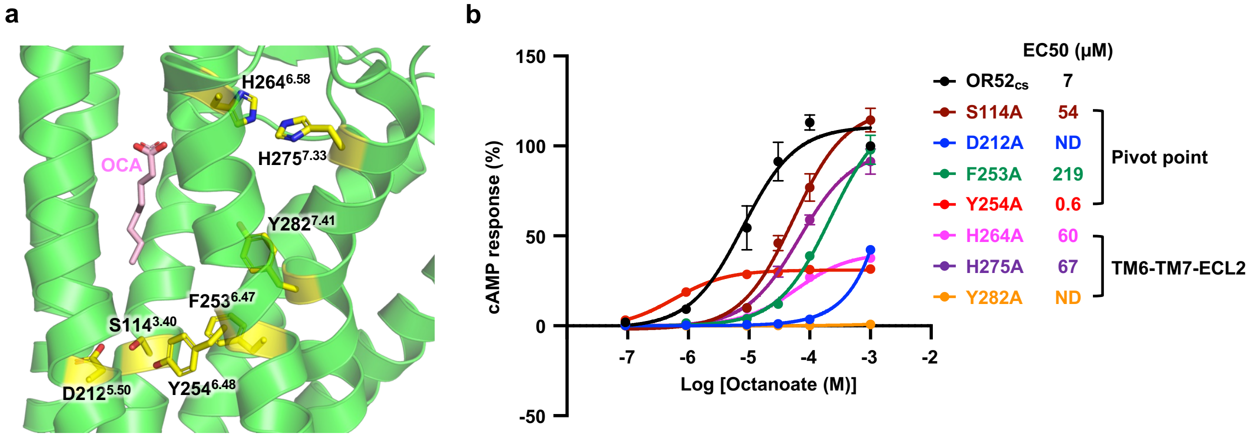


**Supplementary Fig. 13| Mutagenesis of residues important for activation of OR52$_{cs}$. a.** Residues which are not directly involved in odorant binding but stabilize active state are shown as yellow sticks. **b.** cAMP response curve of alanine mutants for residues highlighted in **a**. For each data, symbols and error bars indicate the mean and the standard error of the mean (S.E.M.) from n=3 (for OR52$_{cs}$, n=5) independent experiments, respectively.

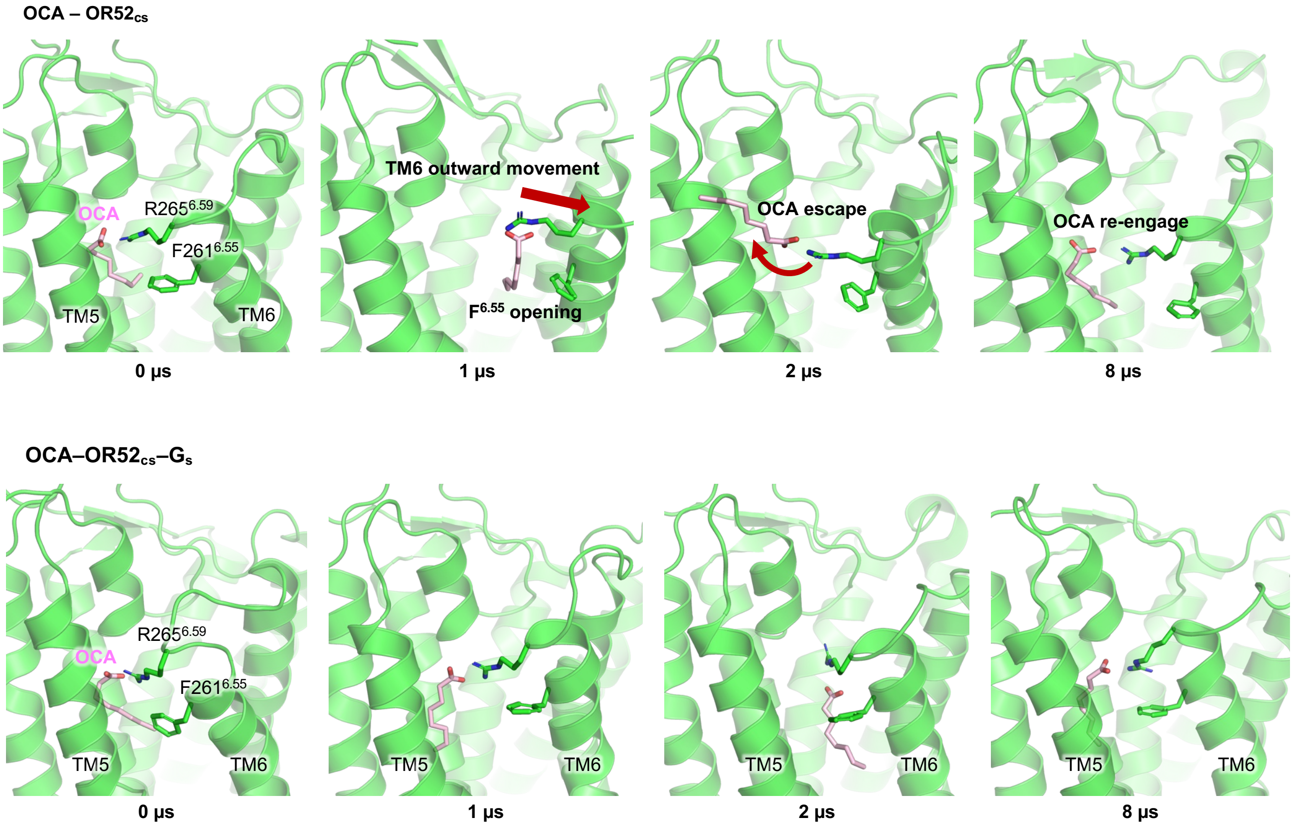


**Supplementary Fig. 14| MD simulations of OCA–OR52$_{cs}$ with or without G$_s$.** Snapshots at 0, 1, 2, and 8 μs are presented for each simulation, with OCA, F261$^{6.55}$, and R265$^{6.59}$ shown as sticks.

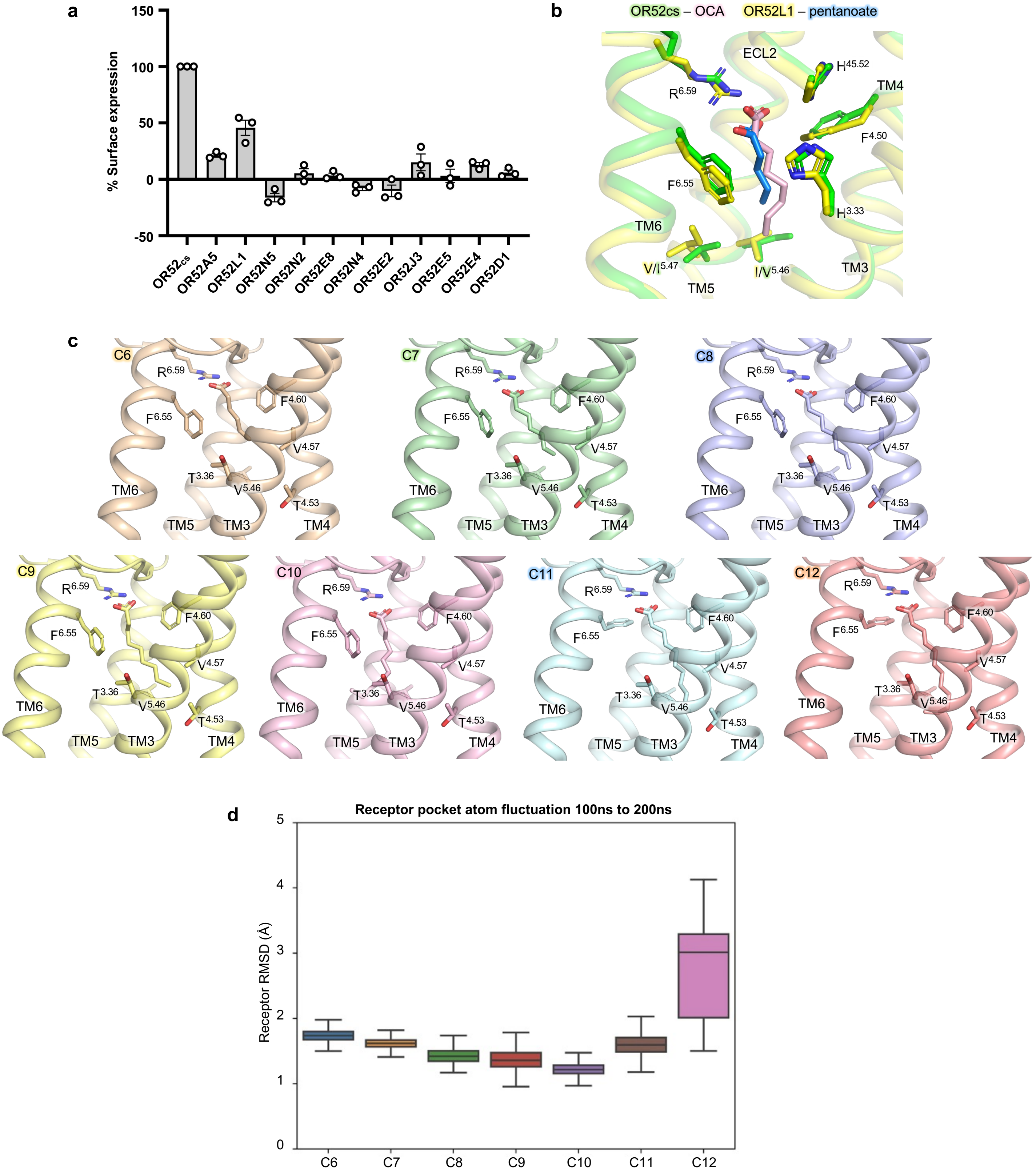


**Supplementary Fig. 15| Structural modeling of pentanoate–OR52L1 and OR52E5 with fatty acids of different lengths. a** Quantification of surface expression of native OR52 family members by surface ELISA. For each data, error bars indicate the standard error of the mean (S.E.M.) of three independent experiments, respectively, and symbols indicate individual values. **b** Structural alignment between OCA–$OR52_{cs}$ and pentanoate–OR52L1 model. **c** Modeling of carboxylate (C6 to C12) bound OR52E5. **d** Fluctuations of the pocket residues in each carboxylate-bound OR52E5 model during 100-200 ns MD simulations are displayed.

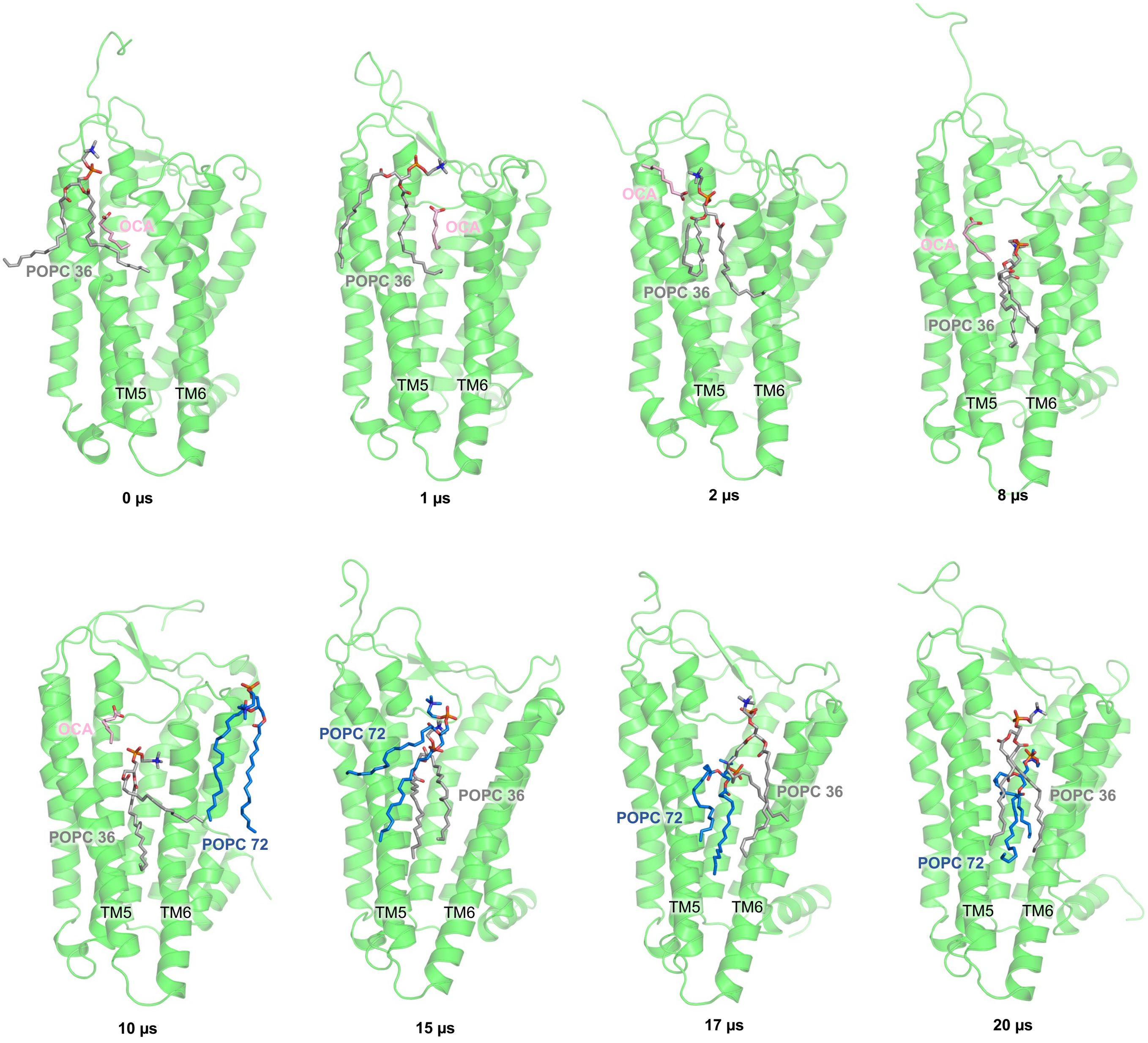


**Supplementary Fig. 16| Entrance and exchange of phospholipids during 20 μs MD simulation of OCA–OR52$_{cs}$.** Entrance of phospholipid (POPC36) into OR52$_{cs}$ through the opening between TM5 and TM6 was observed in 2 μs simulation. After the total escape of OCA, another phospholipid (POPC72) entered OR52$_{cs}$ while pushing POPC36 out in 17 μs. The presence of POPC72 in OR52$_{cs}$ was observed until the end of the simulation (20 μs).

**Supplementary Table. 1| Crystallographic statistics of $G\alpha_{olf}$**

| | |
|---|---|
| *Data collection* | |
| wavelength (Å) | 0.9794 |
| Space group | $P2_1$ |
| Unit cell parameters (a, b, c, β) | 121.7 Å, 52.4 Å, 125.5 Å, 118.5° |
| Resolution (Å) (last shell) | 27 - 2.9 (3.0 - 2.9) |
| Unique reflections | 30932 (3030) |
| Completeness (%) | 99.4 (99.8) |
| Multiplicity | 3.4 (3.5) |
| I/σ(I) | 7.1 (1.5) |
| $R_{merge}{}^{a}$ | 0.178 (0.808) |
| $CC_{1/2}{}^{b}$ | 0.983 (0.67) |
| *Refinement* | |
| No. of reflections working set (test set) | 30932 (1547) |
| $R_{work}/ R_{free}{}^{c}$ | 0.23 / 0.27 |
| bond length rmsd from ideal (Å) | 0.002 |
| bond angle rmsd from ideal (°) | 0.56 |
| Ramachandran analysis[d] | |
| % favored regions | 98.02 |
| % allowed regions | 1.98 |
| % outliers | 0 |

[a] $R_{merge}$=ΣhΣI|Iih<Ih>|ΣhΣi(h), where Ii(h) is the ith measurement of reflection h, and <I(h)> is the weighted mean of all measurements of h.

[b]$CC_{1/2}$ : Pearson correlation coefficient between random half-datasets[10].

[c]$R = \Sigma_h|F_{obs}(h)| - |F_{calc}(h)| \;|\; / \;\Sigma_h|F_{obs}(h)|$. $R_{work}$ and $R_{free}$ were calculated using the working and test reflection sets, respectively.

[d]As defined in MolProbity[11].

**Supplementary Table. 2| Cryo-EM data collection, refinement and validation statistics**

| | OCA–OR52$_{cs}$–G$_s$–Nb35 | Apo state OR52$_{cs}$ | |
|---|---|---|---|
| Composite map | EMD-35010 | EMD-35971 | |
| Consensus map | EMD-35770 | | |
| OR52$_{cs}$-focused map | EMD-35772 | | EMD-37336 |
| G$_s$-focused map | EMD-35773 | | |
| | | With bRIL | No bRIL |
| PDB | PDB 8HTI | PDB 8J46 | PDB 8W77 |
| **Data collection and processing** | | | |
| Magnification | 105,000 | 105,000 | |
| Voltage (kV) | 300 | 300 | |
| Electron exposure (e–/Å$^2$) | 60 | 68.5 | |
| Defocus range (μm) | -0.8 to -2.0 | -0.7 to -1.9 | |
| Pixel size (Å) | 0.851238 | 0.848 | |
| Symmetry imposed | C1 | C1 | |
| Initial particle images (no.) | 493,826 | 5,594,893 | |
| Final particle images (no.) | 126,896 | 173,732 | 142,861 |
| Map resolution (Å) | 2.97 | 3.66 | 3.60 |
| FSC threshold | 0.143 | 0.143 | 0.143 |
| **Refinement** | | | |
| Initial model used (PDB code) | Alphafold2, 3SN6 | Alphafold2 | Alphafold2, 6WW2 |
| Model resolution (Å) | 3.10 | 3.80 | 3.50 |
| FSC threshold | 0.143 | 0.143 | 0.143 |
| Model resolution range (Å) | n/a | n/a | n/a |
| Map sharpening *B* factor (Å$^2$) | -77.6 | -124.9 | -136.3 |
| Model composition | | | |
| Non-hydrogen atoms | 7,393 | 2,158 | 1,601 |
| Protein residues | 962 | 362 | 260 |
| Ligands | OCA: 1 | n/a | n/a |
| B factors (Å$^2$) | | | |
| Protein | 49.15 | 181.08 | 170.24 |
| Ligand | 42.68 | n/a | n/a |
| R.m.s. deviations | | | |
| Bond lengths (Å) | 0.004 | 0.005 | 0.005 |
| Bond angles (°) | 0.600 | 1.133 | 1.141 |
| Validation | | | |
| MolProbity score | 1.50 | 0.68 | 0.73 |
| Clashscore | 5.94 | 0.54 | 0.72 |
| Poor rotamers (%) | 0.00 | 0.00 | 0.00 |
| Ramachandran plot | | | |
| Favored (%) | 97.02 | 99.14 | 99.19 |
| Allowed (%) | 2.98 | 0.86 | 0.81 |
| Disallowed (%) | 0.00 | 0.00 | 0.00 |

| $OR52_{cs}$ Mutants | Octanoate (OCA) | | | Surface Expression (%) |
|---|---|---|---|---|
| | EC50 (μM) (pEC50 ± SEM) | N | X-fold over $OR52_{cs}$ | |
| $OR52_{cs}$ | 7.9 (5.230 ± 0.107) | 5 | 1 | 100 |
| $H107^{3.33}A$ | 187 (2.380 ± 0.092) | 3 | 23.7 | 91.7 ± 9.3 |
| $T110^{3.36}A$ | 22.7 (4.293 ± 0.098) | 3 | 2.9 | 89.0 ± 3.5 |
| $F161^{4.60}A$ | 42.0 (4.214 ± 0.075) | 3 | 5.3 | 110.6 ± 3.5 |
| $H183^{45.52}A$ | 41.4 (4.154 ± 0.087) | 3 | 3.2 | 112.3 ± 3.0 |
| $G201^{5.39}A$ | ND | 3 | - | 116.4 ± 6.8 |
| $I208^{5.46}A$ | 5.1 (5.321 ± 0.119) | 3 | 0.65 | 94.2 ± 3.9 |
| $F261^{6.55}A$ | 506 ( 3.326 ± 1.547) | 3 | 64.0 | 132.6 ± 12.1 |
| $R265^{6.59}A$ | ND | 3 | - | 163.7 ± 7.2 |
| ΔN(1-18) | ND | 3 | - | 25.6 ± 7.1 |
| F14A/F96A /F170A | 208 | 3 | 26.3 | 63.7 ± 4.0 |

**Supplementary Table. 3| $EC_{50}$ of $OR52_{cs}$ and mutants for octanoate.** $EC_{50}$ (pEC50 ± SEM) values of $OR52_{cs}$ and mutants were measured by cAMP assay with octanoate (OCA) as an odorant. Dose-response curves for each dataset are presented in **Figure 3d**. The number of independent experiments for estimating $EC_{50}$ value is displayed in the third column (N). For each mutant, relative fold of $EC_{50}$ over $OR52_{cs}$ is shown in the last column (X-fold over $OR52_{cs}$). Surface expression level of each mutant was quantified by surface ELISA and normalized by $OR52_{cs}$.

| Active state | | | TM6 Residue | Apo state | | |
|---|---|---|---|---|---|---|
| Interaction character | Distance (Å) | Interacting Residue | | Interacting Residue | Distance (Å) | Interaction character |
| Polar (backbone) | 3.2 | I198$^{5.36}$ | R265$^{6.59}$ | I198$^{5.36}$ | No interaction | |
| van der Waals | 4.0 | I198$^{5.36}$ | | I198$^{5.36}$ | | |
| van der Waals | 3.9 | L202$^{5.40}$ | | L202$^{5.40}$ | | |
| Hydrogen bond | 3.9 | H178$^{45.47}$ | H264$^{6.58}$ | H178$^{45.47}$ | No interaction | |
| Hydrogen bond | 2.5 | E182$^{45.51}$ | | E182$^{45.51}$ | | |
| van der Waals | 3.7 | M184$^{45.53}$ | | M184$^{45.53}$ | | |
| Hydrogen bond | 3.2 | H275$^{7.33}$ | | H275$^{7.33}$ | | |
| van der Waals | 3.9 | E182$^{45.51}$ | F261$^{6.55}$ | E182$^{45.51}$ | No interaction | |
| van der Waals | 3.4 | V209$^{5.47}$ | F258$^{6.52}$ | V209$^{5.47}$ | No interaction | |
| van der Waals | 3.6 | Y282$^{7.41}$ | P256$^{6.50}$ | Y282$^{7.41}$ | No interaction | |
| Hydrogen bond | 2.8 | S114$^{3.40}$ | Y254$^{6.48}$ | S114$^{3.40}$ | 3.9 | Hydrogen bond |
| van der Waals | 3.4 | V209$^{5.47}$ | | V209$^{5.47}$ | No interaction | |
| van der Waals | 3.8 | V213$^{5.51}$ | | V213$^{5.51}$ | | |
| van der Waals | 4.0 | T110$^{3.36}$ | F253$^{6.47}$ | T110$^{3.36}$ | No interaction | |
| van der Waals | 4.1 | E113$^{3.39}$ | | E113$^{3.39}$ | | |
| van der Waals | 4.0 | Y282$^{7.41}$ | | Y282$^{7.41}$ | | |
| van der Waals | 3.7 | P286$^{7.45}$ | | P286$^{7.45}$ | 4.0 | van der Waals |
| van der Waals | 4.3 | I216$^{5.54}$ | I250$^{6.44}$ | I216$^{5.54}$ | 3.6 | van der Waals |
| van der Waals | 4.0 | Y220$^{5.58}$ | | Y220$^{5.58}$ | 4.5 | van der Waals |
| van der Waals | 3.3 | N290$^{7.49}$ | V249$^{6.43}$ | N290$^{7.49}$ | 4.3 | van der Waals |
| van der Waals | 3.6 | I293$^{7.52}$ | | I293$^{7.52}$ | 4.4 | van der Waals |
| No interaction | | Y294$^{7.53}$ | | Y294$^{7.53}$ | 3.6 | van der Waals |
| Polar | 2.8 | Y220$^{5.58}$ | H246$^{6.40}$ | Y220$^{5.58}$ | 2.8 | Polar |
| van der Waals | 3.9 | Y294$^{7.53}$ | | Y294$^{7.53}$ | 3.8 | van der Waals |
| van der Waals | 3.6 | Y220$^{5.58}$ | C243$^{6.37}$ | Y220$^{5.58}$ | 3.1 | Hydrogen bond |

**Supplementary Table. 4| Key interactions of TM6 residues stabilizing the apo and active states of OR52$_{cs}$.** Key interactions of TM6 residues stabilizing the apo and active states of OR52$_{cs}$ are compared in this table. Interactions between TM6 residues are not included. For each interaction, the distance (Å) and interaction character are displayed. Residues with no evident side chain are indicated as ‘No interaction’.

| OR species | TM3 | | | TM4 | ECL2 | TM5 | | | | | TM6 | | | Sequence Identity (%) | |
|---|---|---|---|---|---|---|---|---|---|---|---|---|---|---|---|
| | | | | | | | | | | | | | | Pocket residues | Whole residues |
| OR52$_{cs}$ | H107$^{3.33}$ | T110$^{3.36}$ | G111$^{3.37}$ | F161$^{4.60}$ | H183$^{45.52}$ | G201$^{5.39}$ | V204$^{5.42}$ | A205$^{5.43}$ | I208$^{5.46}$ | V209$^{5.47}$ | F258$^{6.52}$ | F261$^{6.55}$ | R265$^{6.59}$ | 100 | 100 |
| OR52N4 | H | T | G | F | H | G | V | A | I | W | F | F | R | 92.31 | 64.01 |
| OR52N5 | H | T | G | F | H | G | V | A | I | G | F | F | R | 92.31 | 63.28 |
| OR52N2 | H | T | G | F | H | G | V | A | I | G | F | F | R | 92.31 | 61.78 |
| OR52E4 | H | T | G | F | H | G | V | I | I | I | F | F | R | 84.62 | 70.61 |
| OR52H1 | H | F | V | D | H | G | V | P | T | V | F | I | R | 84.62 | 60.91 |
| OR52N1 | H | T | G | S | H | G | V | A | I | G | F | F | H | 76.92 | 60 |
| OR52J3 | H | T | G | M | H | G | V | V | F | V | V | F | R | 69.23 | 64.94 |
| OR52A5 | H | Q | A | S | H | G | V | A | I | L | F | F | R | 69.23 | 64.01 |
| OR52D1 | H | Y | A | F | H | G | V | A | A | M | F | F | R | 69.23 | 63.81 |
| OR52E8 | H | T | A | L | H | G | N | I | L | L | F | F | R | 61.54 | 73.16 |
| OR52E5 | H | T | G | F | H | G | A | F | V | G | L | F | R | 61.54 | 66.77 |
| OR52K1 | H | S | I | L | H | G | V | A | I | V | V | S | R | 61.54 | 63.87 |
| OR52K2 | H | S | I | L | H | G | V | A | I | V | V | S | R | 61.54 | 63.26 |
| OR52A1 | H | Q | G | C | H | G | V | A | V | A | F | F | R | 61.54 | 60.97 |
| OR52E6 | H | T | V | L | H | G | S | I | L | L | F | F | C | 53.85 | 69.01 |
| OR52E2 | H | T | L | S | H | G | A | I | L | V | L | F | R | 53.85 | 68.61 |
| OR52R1 | H | S | S | F | H | G | V | A | V | A | L | F | R | 53.85 | 62.62 |
| OR52L1 | H | S | S | F | H | G | M | A | V | I | I | F | R | 53.85 | 61.13 |
| OR52W1 | H | T | A | F | H | G | L | S | I | S | L | Y | R | 53.85 | 49.68 |
| OR52B2 | H | F | V | V | H | G | V | P | M | V | F | L | H | 46.15 | 65.81 |
| OR52B4 | H | F | I | I | H | G | I | L | T | V | I | I | R | 38.46 | 61.34 |
| OR52B6 | H | L | F | S | H | G | A | A | S | T | L | V | R | 38.46 | 60.13 |
| OR52A4 | H | Q | G | C | R | G | G | A | V | G | F | I | Q | 38.46 | 55.38 |
| OR52M1 | H | A | T | L | H | G | I | G | V | L | A | S | R | 30.77 | 59.53 |
| OR52I1 | H | T | A | L | H | S | G | S | M | V | M | I | W | 30.77 | 51.48 |
| OR52I2 | H | T | A | L | H | S | G | S | M | V | M | I | W | 30.77 | 51.48 |

**Supplementary Table. 5| Sequence alignment of residues around odorant binding pocket in the OR52 family.** The sequence alignment of human OR52 family members with OR52$_{cs}$. Residues constituting odorant-binding pocket are presented. Sequence identity for each OR52 family member against OR52$_{cs}$ was presented, both for whole sequence and pocket residues.

**Supplementary Table. 6| Detailed system information of MD simulations.**

| | OCA–OR52$_{cs}$–G$_s$ | OCA–OR52$_{cs}$ | apo OR52$_{cs}$ |
|---|---|---|---|
| Simulation box (Å$^3$) | 139 × 139 × 171 | 91 × 91 × 113 | 90 × 90 × 116 |
| # atoms | 308,264 | 85,555 | 87,389 |
| # water molecules | 221,229 | 17,823 | 18,441 |
| Lipid composition | | POPC:cholesterol (4:1) | |
| Upper leaflet, # lipids, POPC | 228 | 88 | 88 |
| Upper leaflet, # lipids, cholesterol | 57 | 22 | 22 |
| Lower leaflet, # lipids, POPC | 224 | 88 | 88 |
| Lower leaflet, # lipids, cholesterol | 56 | 22 | 22 |
| Salt concentration (M), KCl | | 0.15 | |

## References


1 Olender, T., Nativ, N. & Lancet, D. HORDE: comprehensive resource for olfactory receptor genomics. *Methods Mol. Biol*. **1003**, 23-38 (2013).

2 Crooks, G. E., Hon, G., Chandonia, J. M. & Brenner, S. E. WebLogo: a sequence logo generator. *Genome Res*. **14**, 1188-1190 (2004).

3 Isberg V, *et al*. Generic GPCR residue numbers - aligning topology maps while minding the gaps. *Trends Pharmacol. Sci.* **36**, 22-31 (2015).

4 Sunahara, R. K., Tesmer, J. J., Gilman, A. G. & Sprang, S. R. Crystal structure of the adenylyl cyclase activator Gsalpha. *Science* **278**, 1943-1947 (1997).

5 Punjani, A., Rubinstein, J. L., Fleet, D. J. & Brubaker, M. A. cryoSPARC: algorithms for rapid unsupervised cryo-EM structure determination. Nat. Methods 14, 290-296 (2017).

6 Rubinstein, J. L. & Brubaker, M. A. Alignment of cryo-EM movies of individual particles by optimization of image translations. *J. Struct. Biol*. **192**, 188-195 (2015).

7 Pettersen, E. F. *et al*. UCSF ChimeraX: Structure visualization for researchers, educators, and developers. *Protein Sci.* **30**, 70-82 (2021).

8 Zivanov, J. *et al*. New tools for automated high-resolution cryo-EM structure determination in RELION-3. *eLife* **7**, e42166 (2018).

9 Pintilie, G., Zhang, K., Su, Z., Li, S., Schmid, M. F. & Chiu, W. Measurement of atom resolvability in cryo-EM maps with Q-scores. *Nat. Methods* **17**, 328-334 (2020).

10 Diederichs, K. & Karplus, P. A. Better models by discarding data? *Acta Crystallogr. Sect. D. Biol. Crystallogr*. **69**, 1215-1222 (2013).

11 Chen, V. B. *et al*. MolProbity: all-atom structure validation for macromolecular crystallography. *Acta Crystallogr. Sect. D. Biol. Crystallogr.* **66**, 12-21 (2010).

## [MD simulation checklist]

| Reliability and reproducibility checklist for molecular dynamics simulations<br>*All boxes must be marked YES by acceptance unless "Response not needed if No". | Yes | No | Response<br>(Please state where this information can be found in the text) |
|---|---|---|---|
| **1. Convergence of simulations and analysis** | | | |
| 1a. Is an evaluation presented in the text to show that the property being measured has equilibrated in the simulations (e.g. time-course analysis)? | ☒ | ☐ | In the Methods section, the following sentence has been added with a reference.<br>Line 555: "Following the CHARMM-GUI six-step equilibration procedure," |
| 1b. Then, is it described in the text how simulations are split into equilibration and production runs and how much data were analyzed from production runs? | ☒ | ☐ | In the Methods section, the following sentence has been added with a reference.<br>Line 555: "Following the CHARMM-GUI six-step equilibration procedure," |
| 1c. Are there at least 3 simulations per simulation condition with statistical analysis? | ☒ | ☐ | We used 5 replicas, which can be confirmed in Figures 3e and 4c, as well as in the Methods section (Line 562: "Simulations were performed at least 1 μs for five replicas"). For Anton2 simulation, we conducted single 10 μs and 20 μs all-atom MD simulations for OCA-OR52$_{cs}$–G$_s$ and OCA–OR52$_{cs}$, respectively. Although we conducted extensive simulations for these systems, the sampling may vary when reproduced. |
| 1d. Is evidence provided in the text that the simulation results presented are independent of initial configuration? | ☒ | ☐ | It can be seen in Figures 3e and 4c. |
| **2. Connection to experiments** | | | |
| 2a. Are calculations provided that can connect to experiments (e.g. loss or gain in function from mutagenesis, binding assays, NMR chemical shifts, J-couplings, SAXS curves, interaction distances or FRET distances, structure factors, diffusion coefficients, bulk modulus and other mechanical properties, etc.)? | ☒ | ☐ | Calculations (Figure 3e) can be connected to reduced cAMP responses in Figure 3d. |
| **3. Method choice** | | | |
| 3a. Do simulations contain membranes, membrane proteins, intrinsically disordered proteins, glycans, nucleic acids, polymers, or cryptic ligand binding? | ☒ | ☐ | Simulations contain membranes and membrane proteins as described In the Methods section.<br>Line 544: "The receptor was embedded into a model membrane composed of 1-palmitoyl-2-oleoyl-sn-glycero-3-phosphocholine (POPC) and cholesterol (4:1)." |
| 3b. Is it described in the text whether the accuracy of the chosen model(s) is sufficient to address the question(s) under investigation (e.g. all-atom vs. coarse-grained models, fixed charge vs. polarizable force fields, implicit vs. explicit solvent or membrane, force field and water model, etc.)? | ☒ | ☐ | Force fields that are employed in this study were described in the Methods section.<br>Line 549: "The CHARMM36(m) force field was utilized for lipids and proteins, and CGenFF was used for the OCA ligand."<br>For a better understanding, we have changed "MD simulations" into "All-atom MD simulations" in the text. |
| 3c. Is the timescale of the event(s) under investigation beyond the brute-force MD simulation timescale in this study that enhanced sampling methods are needed? | ☐ | ☒ | MD simulation in this study does not require enhanced sampling methods. |
| If YES, are the parameters and convergence criteria for the enhanced sampling method clearly stated? | ☐ | ☐ | |
| If NO, is the evidence provided in the text? | ☒ | ☐ | We performed free MD simulations, and the details are addressed in the Methods section.<br>Line 562: "Simulations were performed at least 1 μs for five replicas using OpenMM simulation package."<br>Line 564: "longer time scale up to 20 μs and 10 μs for OCA–OR52$_{cs}$ and OCA–OR52$_{cs}$–G$_s$, respectively." |
| **4. Code and reproducibility** | | | |
| 4a. Is a table provided describing the system setup that includes simulation box dimensions, total number of atoms, total number of water molecules, salt concentration, lipid composition (number of molecules and type)? | ☒ | ☐ | We have properly updated the information in the Methods section and provided a table (Supplementary Table 6).<br>Line 541: "Three model systems were prepared for all-atom MD simulation: OCA–OR52$_{cs}$–G$_s$ (139 × 139 × 171 Å$^3$), OCA–OR52$_{cs}$ (91 × 91 × 113 Å$^3$), and Apo OR52$_{cs}$ (90 × 90 × 116 Å$^3$), with the total numbers of atoms (and water molecules) of 308,264 (221,229), 85,555 (17,823), and 87,389 (18,441), respectively (Supplementary Table 6)." |
| 4b. Is it described in the text what simulation and analysis software and which versions are used? | ☒ | ☐ | Described in the Methods section, "All-atom MD simulations of apo and OCA-bound states of OR52$_{cs}$".<br>Line 562: "Simulations were performed at least 1 μs for five replicas using OpenMM simulation package." |
| 4c. Are other parameters for the system setup described in the text, such as protonation state, type of structural restraints if applied, nonbonded cutoff, thermostat and barostat, etc.? | ☒ | ☐ | Described in the Methods section, "All-atom MD simulations of apo and OCA-bound states of OR52$_{cs}$".<br>Line 545: "For the G protein, three lipidations were introduced into the Gα$_s$ and Gγ proteins, i.e., N-myristoylation (Gly2 of Gα$_s$), S-palmitoylation (Cys3 of Gα$_s$), and S-geranylgeranylation (C68 of Gγ)." |
| 4d. Are initial coordinate and simulation input files and a coordinate file of the final output provided as supplementary files or in a public repository? | ☒ | ☐ | We have provided a link.<br>Line 601: "The initial and final configurations obtained from 1-μs all-atom MD simulations and extended simulations from Anton2 of all model systems are available at https://github.com/sek24/natcomm2023." |
| 4e. Is there custom code or custom force field parameters? | ☐ | ☒ | Response not needed if No |
| If YES, are they provided as supplementary files or in a public repository? | ☐ | ☐ | |

## <Uncropped gel for Supplementary Figure. 3a>

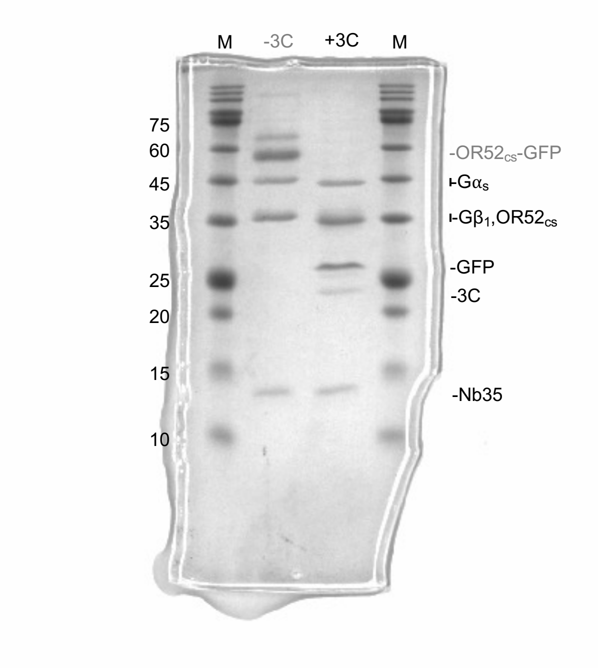


## <Uncropped gel for Supplementary Figure. 10a>

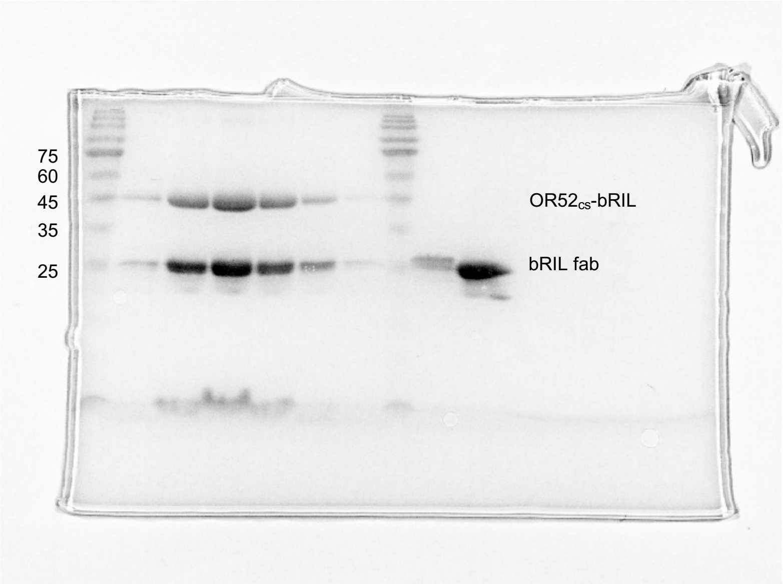

Supplement: Supplementary file 1 — Supplementary Information [file 41467_2023_43983_MOESM1_ESM.pdf]
