# Supplementary material for: Understanding the molecular mechanisms of odorant binding and activation of the human OR52 family
Source: Nat Commun. 2023 Dec 7;14:8105. doi: 10.1038/s41467-023-43983-9 (PMC10703812; doi:10.1038/s41467-023-43983-9)
Supplement: Supplementary file 3 — Reporting Summary [file 41467_2023_43983_MOESM3_ESM.pdf]

nature portfolio

Corresponding author(s): Hee-jung Choi

Last updated by author(s): Nov 20, 2023

Reporting Summary

Nature Portfolio wishes to improve the reproducibility of the work that we publish. This form provides structure for consistency and transparency in reporting. For further information on Nature Portfolio policies, see our Editorial Policies and the Editorial Policy Checklist.

Statistics

For all statistical analyses, confirm that the following items are present in the figure legend, table legend, main text, or Methods section.

n/a	Confirmed	
☐	☒	The exact sample size (*n*) for each experimental group/condition, given as a discrete number and unit of measurement
☐	☒	A statement on whether measurements were taken from distinct samples or whether the same sample was measured repeatedly
☒	☐	The statistical test(s) used AND whether they are one- or two-sided<br>*Only common tests should be described solely by name; describe more complex techniques in the Methods section.*
☒	☐	A description of all covariates tested
☐	☒	A description of any assumptions or corrections, such as tests of normality and adjustment for multiple comparisons
☐	☒	A full description of the statistical parameters including central tendency (e.g. means) or other basic estimates (e.g. regression coefficient) AND variation (e.g. standard deviation) or associated estimates of uncertainty (e.g. confidence intervals)
☒	☐	For null hypothesis testing, the test statistic (e.g. *F*, *t*, *r*) with confidence intervals, effect sizes, degrees of freedom and *P* value noted<br>*Give P values as exact values whenever suitable.*
☒	☐	For Bayesian analysis, information on the choice of priors and Markov chain Monte Carlo settings
☒	☐	For hierarchical and complex designs, identification of the appropriate level for tests and full reporting of outcomes
☐	☒	Estimates of effect sizes (e.g. Cohen's *d*, Pearson's *r*), indicating how they were calculated

Our web collection on statistics for biologists contains articles on many of the points above.

Software and code

Policy information about availability of computer code

Data collection	A diffraction data set of human olfactory G alpha subunit was collected at beamline 5C at the Pohang Accelerator Laboratory (PAL, KOREA). Data collection and refinement statistics are shown in Supplementary Table 1. Cyro-EM data of the OR52cs-bRIL-Fab complex and OCA-OR52cs-Gs-Nb35 complex were collected at the Institute of Basic Science (IBS, KOREA) and the Institute of Membrane Proteins (IMP, Korea), respectively. Cryo-EM data collection and refinement statistics are shown in Supplementary Table 2.
Data analysis	The apo and active OR52cs data sets were motion-corrected, CTF estimation, 2D-averaging, 3D-refinement and local refinement using cryoSPARC 4.2.0 and cryoSPARC 3.3.2, respectively. Additionally, Topaz 0.2.4. for particle picking, RELION 3.1.1, MapQ 1.9.9, Coot 0.9.4, Phenix 1.19.1, MolProbity 4.5, XDS, Chimera 1.15, ChimeraX 1.2.5 and 1.6.1, Pymol 2.5.1, GraphPad Prism 9.4.1, MAFFT 7.475, Weblogo3, MEGAX 10.2.5, OpenMM, Amber20, CHARMM-GUI 3.6, Rosetta GALigandDock, Rosetta FastRelax, AlphaFold2 were used in this study.

For manuscripts utilizing custom algorithms or software that are central to the research but not yet described in published literature, software must be made available to editors and reviewers. We strongly encourage code deposition in a community repository (e.g. GitHub). See the Nature Portfolio guidelines for submitting code & software for further information.

Data

Policy information about availability of data

All manuscripts must include a data availability statement. This statement should provide the following information, where applicable:

- Accession codes, unique identifiers, or web links for publicly available datasets
- A description of any restrictions on data availability
- For clinical datasets or third party data, please ensure that the statement adheres to our policy

Final model and structure factor for the structure of olfactory G alpha protein are deposited in Protein Data Bank under accession number of 8HTG. The cryo-EM density map for the OCA-OR52cs-Gs-Nb35 complex and apo state OR52cs and OR52cs-bRIL have been submitted in the Electron Microscopy Data Bank (EMDB) under accession numbers of EMD-35010, EMD-35770, EMD-35772, EMD-35773, EMD-37336, and EMD-35971. And, their coordinates are deposited in Protein Data Bank under accession numbers of 8HTI, 8WW7, and 8J46, respectively. They will be released upon publication. Structural models used in data analysis were accessed from the Protein Data Bank under the accession number of 6WW2, 1AZT, 8F76, 3SN6, 6VMS, 6DDF, 2RH1, and 6LI1. Other data supporting this work are available from the corresponding author upon reasonable request. Source data are provided with this paper.

Research involving human participants, their data, or biological material

Policy information about studies with human participants or human data. See also policy information about sex, gender (identity/presentation), and sexual orientation and race, ethnicity and racism.

Reporting on sex and gender	Not relevant to this study.
Reporting on race, ethnicity, or other socially relevant groupings	Not relevant to this study.
Population characteristics	Not relevant to this study.
Recruitment	Not relevant to this study.
Ethics oversight	Not relevant to this study.

Note that full information on the approval of the study protocol must also be provided in the manuscript.

Field-specific reporting

Please select the one below that is the best fit for your research. If you are not sure, read the appropriate sections before making your selection.

☒ Life sciences ☐ Behavioural & social sciences ☐ Ecological, evolutionary & environmental sciences

For a reference copy of the document with all sections, see nature.com/documents/nr-reporting-summary-flat.pdf

Life sciences study design

All studies must disclose on these points even when the disclosure is negative.

Sample size	There are no general statistical methods for determining sample size in cryo-EM and X-ray crystallography. Proper data sets were collected to ensure unambiguous model building in crystallography density map and cryo-EM density map. For cryo-EM study, the number of particles that went into the refined map was determined via 3D classification, as described in Methods. For functional studies, multiple experiments were performed and the number of experiments is described in each figure legend. The sample size of functional studies was determined to be more than 3 to ensure statistical reliabiliy.
Data exclusions	Cryo-EM data were pre-processed in CryoSPARC and low-quality micrograpahs (highly contaminated (ice or ethane) and low estimated CTF, high stage drift micrographs) were excluded using statistical methods; these are common exclusion criteria in cryo-EM data analysis. No data were excluded in other functional assays.
Replication	At least three or more independent replicates of each BRET assay (Supplementary Figure. 2), cAMP assay (except for T110A; two replicates) (Figure. 1-3, Supplementary Figure. 8, 11, 13, and Supplementary Table 3), CRE luciferase assay (Supplementary Figure. 7), and ELISA-based surface expression assay (Extended data Fig. 15, and Supplementary Table 3) were performed. Five replica (Figures 3, 4) and three replica (Extended data Fig. 15) of MD simulations were carried out. Functional studies were replicated within several months and replications were successful and consistent.
Randomization	No randomization was performed. Single particle cryo-EM analysis based on randomly oriented and distributed particles in grid hole. For 3D reconstruction, particle set were randomly assigned to calculate gold-standard FSC. For functional studies, conditions such as detection position in well-plate were randomly assigned for each constructed tested.
Blinding	Blinding is not relevant to this study. Protein samples and cultured cell lines are not required to be allocated into experimental groups

Reporting for specific materials, systems and methods

We require information from authors about some types of materials, experimental systems and methods used in many studies. Here, indicate whether each material, system or method listed is relevant to your study. If you are not sure if a list item applies to your research, read the appropriate section before selecting a response.

Materials & experimental systems

n/a	Involved in the study
☐	☒ Antibodies
☐	☒ Eukaryotic cell lines
☒	☐ Palaeontology and archaeology
☒	☐ Animals and other organisms
☒	☐ Clinical data
☒	☐ Dual use research of concern
☒	☐ Plants

Methods

n/a	Involved in the study
☒	☐ ChIP-seq
☒	☐ Flow cytometry
☒	☐ MRI-based neuroimaging

Antibodies

Antibodies used	Rabbit anti-FLAG monoclonal antibody (Cell signaling Technology, D6W5B), Goat anti-rabbit IgG polyclonal antibody HRP conjugate (Enzo Life Sciences, ADI-SAB-300-J)
Validation	The primary anti-FLAG monoclonal antibody (Cell signaling Technology, D6W5B) was validated by manufacturer's website, using GFP-DYKDDDDK control protein detection.

Eukaryotic cell lines

Policy information about cell lines and Sex and Gender in Research

Cell line source(s)	Sf9 insect cells (Expression Systems, 94-00lF), High Five insect cells (Expression Systems, 94-002F), Hana3A (Matsunami Lab, Cellosaurus ID: CVCL_RW32), Rosetta (Novagen)
Authentication	The cell lines were authenticated by the manufactures based on the manufactures' descriptions. After purchase, Sf9, high five, and rosetta cell lines were not authenticated by our group. The Matsunami Lab was responsible for the generation and authentication of the Hana3A cell line. RT-PCR was used to authenticate HANA3A cell line by Matsunami Lab's work in 2004 (DOI: 10.1016/j.cell.2004.11.021)
Mycoplasma contamination	Hana3A, Sf9, Hi5, and rosetta cell line was not tested for mycoplasma contamination.
Commonly misidentified lines (See ICLAC register)	No commonly misidentified cells were used in this study.

Plants

Seed stocks	Not relevant to this study.
Novel plant genotypes	Not relevant to this study.
Authentication	Not relevant to this study.
